# Ciguatera Fish Poisoning in the Caribbean Sea and Atlantic Ocean: Reconciling the Multiplicity of Ciguatoxins and Analytical Chemistry Approach for Public Health Safety

**DOI:** 10.3390/toxins15070453

**Published:** 2023-07-10

**Authors:** Ivannah Pottier, Richard J. Lewis, Jean-Paul Vernoux

**Affiliations:** 1 Normandie Univ, UNICAEN, UNIROUEN, ABTE, 14000 Caen, France; ivannah.pottier@unicaen.fr; 2 Institute for Molecular Bioscience, The University of Queensland, Brisbane 4072, Australia

**Keywords:** ciguatera fish poisoning, Caribbean ciguatoxins, Caribbean Sea and Atlantic Ocean, fish toxins, marine biotoxins, phycotoxins, *Gambierdiscus*, ciguatoxins isolation, mass spectrometry analysis, LC-MS, LC-HRMS, food safety

## Abstract

Ciguatera is a major circumtropical poisoning caused by the consumption of marine fish and invertebrates contaminated with ciguatoxins (CTXs): neurotoxins produced by endemic and benthic dinoflagellates which are biotransformed in the fish food-web. We provide a history of ciguatera research conducted over the past 70 years on ciguatoxins from the Pacific Ocean (P-CTXs) and Caribbean Sea (C-CTXs) and describe their main chemical, biochemical, and toxicological properties. Currently, there is no official method for the extraction and quantification of ciguatoxins, regardless their origin, mainly due to limited CTX-certified reference materials. In this review, the extraction and purification procedures of C-CTXs are investigated, considering specific objectives such as isolating reference materials, analysing fish toxin profiles, or ensuring food safety control. Certain in vitro assays may provide sufficient sensitivity to detect C-CTXs at sub-ppb levels in fish, but they do not allow for individual identification of CTXs. Recent advances in analysis using liquid chromatography coupled with low- or high-resolution mass spectrometry provide new opportunities to identify known C-CTXs, to gain structural insights into new analogues, and to quantify C-CTXs. Together, these methods reveal that ciguatera arises from a multiplicity of CTXs, although one major form (C-CTX-1) seems to dominate. However, questions arise regarding the abundance and instability of certain C-CTXs, which are further complicated by the wide array of CTX-producing dinoflagellates and fish vectors. Further research is needed to assess the toxic potential of the new C-CTX and their role in ciguatera fish poisoning. With the identification of C-CTXs in the coastal USA and Eastern Atlantic Ocean, the investigation of ciguatera fish poisoning is now a truly global effort.

## 1. Introduction

Ciguatera fish poisoning (CP) is a seafood-borne illness that is endemic to many tropical and subtropical regions of the world [1,2,3,4,5,6,7]. It is caused by the ingestion of a range of fish species that are generally associated with coral reefs from all oceans and seas, especially between the latitudes of 35° N and 35° S. Ciguatera can be also caused by the consumption of marine invertebrates such as sea urchins [8], lobsters and octopuses [9,10], giant clams [10,11], and sea stars [12]. Authorities and researchers have published rapid fish identification guides with colour photographs to enable the general public to identify fishes in the region that may pose a risk of ciguatera [13,14,15]. Despite these precautions, ciguatera risk remains poorly managed, with exposure to potentially ciguatoxic fishes growing as the international fish trade increases [7,16,17]. Some countries or regions use reporting systems, for example, the surveillance system of the Centers for Disease Control and Prevention (CDC, Atlanta, GE, USA) and the Ciguawatch online site from the Louis Malardé Institute in French Polynesia [18], to track reported cases. In the EU, the Rapid Alert System for Food and Feed (RASFF, European Union) issues alerts to food safety agencies and consumers regarding ciguatera.

In humans, CP is characterized by a polymorphic syndrome with more than 175 symptoms reported by the Food and Agriculture Organization of the United Nations (FAO) and the World Health Organization (WHO) Expert Meeting on Ciguatera Poisoning [19]. Within 24 h following toxic fish consumption, CP usually begins with gastrointestinal symptoms including vomiting, diarrhea, abdominal pain, and nausea, followed by general and neurological effects including cold allodynia, paresthesia in the extremities, a metallic taste, the sensation of loose teeth, pruritus, myalgia, arthralgia, headache, and dizziness [2,3,5,7,16,20,21,22,23]. In the Pacific Ocean, acute neurological forms predominate [2,5,16,24], whereas in the Caribbean, gastrointestinal symptoms are more frequently reported during the early phase of the illness, again followed by neurological signs [2,3,25,26]. Additional hallucinatory symptoms are frequently observed in the Indian Ocean [27]. In more severe cases, cardiovascular symptoms may also occur, with bradycardia and/or hypotension predominating [25,26,28], especially in hospitalized cases [25]. While gastrointestinal and cardiovascular symptoms remain for 1–2 days and 3–4 days, respectively, peripheral neurological symptoms and generalized malaise, weakness, fatigue, and headaches can last for weeks, months, or even years [2,3]. Some ciguatera-specific symptoms (pruritus and cold allodynia), can be exacerbated by alcohol ingestion, and may reappear even in asymptomatic patients after eating non-toxic seafood, caffeine, nuts, chicken, and pork [2,3].

CP-related toxins (CTXs) are closely related structural congeners with a polyether backbone similar to brevetoxins. CTXs are classified based on their geographic origin into Pacific Ocean ciguatoxins (P-CTXs) [29,30,31,32,33,34], Caribbean Sea ciguatoxins (C-CTXs) [3,35,36,37,38,39,40,41,42,43], and Indian Ocean ciguatoxins (I-CTXs) [44,45,46,47]. I-CTX-1 and -2 have the same molecular weight of 1140.6 Da as C-CTX-1 and -2 and are pharmacologically related, but they do not co-elute, and there are no structures available for I-CTX due to poor chromatographic recoveries [44,45,46,47]. Although CP has been known for centuries [48,49], it was only in the late 1950s that several groups of researchers initiated multidisciplinary work to identify the cause of CP and the nature of the causative toxins [50]. These included Albert H. Banner et al. in Hawaii [51,52]), Yoshiro Hashimoto et al. in Japan [53], Takeshi Yasumoto and Paul Scheuer in Hawaii [54], and Jack Randall in Miami [55,56]. In a study of the food chain in the Pacific and the Caribbean [55], Randall showed that carnivorous fish were always more toxic than herbivorous, detritus, and mollusc-feeding fishes. A detailed analysis of the stomach and intestinal contents of surgeonfishes and parrotfishes led him to propose that a benthic organism, most likely a blue-green algae, could be the source of the toxins in CP. The later identification of ciguatoxicity in the muscle, liver, and viscera of the herbivorous surgeonfish *Ctenochaetus striatus* in Tahiti supported this hypothesis when Dr Raymond Bagnis began researching ciguatera in French Polynesia in the late 1960s [57]. In 1975, Bagnis and Vernoux found low levels of ciguatoxins in the muscles and viscera of the surgeonfish *Ctenochaetus striatus* and the grouper *Plectropomus leopardus* [58], using a rapid semi-purification of liposoluble residue known as rapid cold alkali treatment [59]. This confirmed that edible coral fishes can harbour low levels of ciguatoxins, supporting its accumulation along the food web, as predicted by Randall in 1958 [55]. Their rapid cold alkali treatment allows for defatting using saponification at room temperature of any lipid-soluble residue (LR) solubilized in one volume of 5% KOH in 90% ethanol/water. After 5 min, three volumes of water are added, and the ethanolic fraction is extracted using diethyl ether to give an unsaponifiable fraction containing P-CTXs [59].

A collaboration between Japan and French Polynesia showed that corallivorous *Scarus gibbus* and carnivorous *Epinephelus microdon* could also retain ciguatoxins [60,61]. Bagnis and his team (Drollet J., Chungue E., Chanteau S., Thevenin S., Vernoux J.-P.) also studied coral covered with a greenish layer collected in the ciguatera-endemic Gambier Islands of French Polynesia. These coral samples were extracted by Vernoux using acetone in the late 1975, and the dried extract was directly injected into mice or injected after rapid cold alkali treatment [59]. Importantly, these injected mice showed typical specific ciguatoxic signs, including penile symptoms [59], which provided the first evidence that this layer was the source of ciguatoxins [62]. To build on these new findings, the South Pacific Commission (SPC) funded a new mission between the researchers in Tahiti (French Polynesia) and Yasumoto in 1976 to isolate the causative organism of ciguatera. Bagnis et al. and Yasumoto et al. [63,64] further characterised the implicated dinoflagellate, which was later named *Gambierdiscus toxicus* by Adachi and Fukuyo [65,66]. In parallel, Yasumoto first described maitotoxin (MTX), a potent water-soluble toxin found into the gut contents of the surgeonfish *C. striatus* (called “maito” in Tahiti) [67]. Additional experiments initiated in 1975 and 1976 on coral substrate and the viscera of parrotfishes and surgeonfishes suggested that maitotoxin was a marker of the dinoflagellate rather than a precursor of ciguatoxins [62,68]. This is in line with the observation that different strains of *G. toxicus* also produce chemically distinct but pharmacologically related MTXs [32,69,70,71,72,73,74,75,76,77]. Vernoux and Lejeune also suggested that ciguatoxin production may require an enhancer, which could be another microorganism (e.g., bacteria) associated with the dinoflagellate and/or dead coral [62,68]. This hypothesis was supported by experimental results [78,79], and it is in line with the fact that, to our knowledge, gambiertoxin/ciguatoxin production has not been confirmed in axenic cultures. Genetics were also suspected to explain the low percentage of culturable clonal strains of *Gambierdiscus* that produce detectable levels of gambiertoxins/ciguatoxins [77,80]. Significant strain-dependent differences in the levels and types of ciguatoxins and maitotoxins produced by the same *Gambierdiscus* species were highlighted [81]. While *Gambierdiscus* remains the principle genus producing ciguatoxins in the Pacific Ocean, with *Gambierdiscus polynesiensis* confirmed as a ciguatoxin producer [79,82,83,84,85,86], other dinoflagellate species of the genus *Fukuyoa* might also contribute to ciguatera risk in the Atlantic Ocean [81,87,88]. These less polar ciguatoxins, originally referred to as gambiertoxins, initiate upstream contamination of the ciguatera food chain [1,75,89]. Furthermore, they are primarily transferred and metabolized into more polar ciguatoxins through different trophic levels [1,77] to generate species-specific profiles of ciguatoxins [23,42,43,90,91]. In the marine food chain, CTXs are known to undergo bioaccumulation and concentration [16,51,58,59,64,92,93]. While fishes at lower trophic levels, such as herbivorous and detritivores fishes and invertebrates feeders, generally exhibit lower CTX concentrations, those at higher trophic levels, such as carnivorous fishes, tend to have higher concentrations [58,68,94,95]. This transfer and accumulation process has been quantified in recent studies conducted by Holmes et al. [75,89].

The P-CTXs are well-known and well-studied toxins, both chemically and pharmacologically [20,96,97]. However, there is a general lack of knowledge regarding C-CTXs. Isolation and characterisation of P-CTXs were successfully in the 1990s [98,99,100]. Caribbean ciguatoxin was first isolated in 1994 from locally caught barracuda (*Sphyraena barracuda*) and horse-eye jack (*Caranx latus*) [101,102]. The structures of C-CTX-1 and its epimer, C-CTX-2 were elucidated using pure C-CTX-1 isolated from the liver, viscera, and flesh of horse-eye jack (*C. latus*), a fish often implicated in ciguatera fish poisoning and collected from Saint-Barthélemy (Lesser Antilles) in the Eastern Caribbean Sea [35,38]. The multiplicity of C-CTXs analogues was first identified using chromatographic behavior [103] and/or the mouse bioassay (MBA), with fast-acting ciguatoxins (FATs) in addition to classical slower-acting ciguatoxins [104]. However, due to insufficient quantity, FATs have not been definitively characterised [19,23,38,41]. Recently, the likely algal precursor of C-CTX-1/-2 was identified in *Gambierdiscus silvae* and *Gambierdiscus caribaeus* strains from the Caribbean [43]. This ketone variant of C-CTX-1/-2 known as C-CTX-5 could be biotransformed in vitro by fish liver microsomes directly to C-CTX-1/-2, the dominant CTX in ciguatoxic fish from the Caribbean. Importantly, C-CTX-5 from *G. silvae* was confirmed to have voltage-gated sodium-channel-specific activity [43], indicating its potential contribution to ciguatera.

Caribbean ciguatoxins have also been identified in the Eastern Atlantic, including the Canary Islands (Spain) and Madeira (Portugal) [105,106,107,108,109,110,111,112,113]. These include CP outbreaks due to the consumption of a 26-kg amberjack (*Seriola rivoliana*) from the Canary Islands in 2004 [105], a 30-kg amberjack (*Seriola* sp.) caught near the Selvagens Islands (Portugal) in 2008 [107], and many ciguatoxin-positive fish from the Madeira and Selvagens Archipelagos [113]. The influence of climate change are expected to bring ciguatera closer to Europe [6,114,115,116,117]. Reflecting this increasing risk in ciguatera in Europe, the remainder of this review provides an update on the chemical and biochemical properties of ciguatoxins in the Atlantic Ocean, and the potential of analytical chemistry to mitigate the public health impacts and advance our knowledge of the origin and biotransformation of C-CTXs.

## 2. Ciguatoxins Structures and Reference Standards

### 2.1. Structures of the Three Main Types of Ciguatoxins

**Figure 1:**
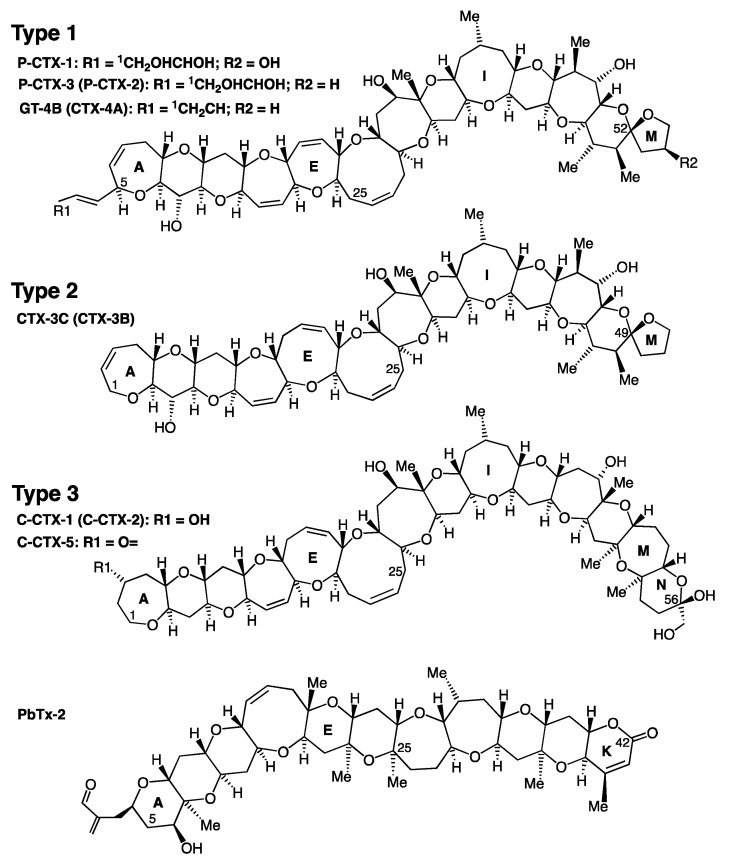
Structures of ciguatoxins from the Pacific Ocean and Caribbean Sea. These structures belong, from top to bottom, to three types of congeners: Type 1 (60 carbons, A to M rings, DEF rings size 7/7/9), Type 2 (57 carbons, A to M rings, DEF rings size 7/8/9), and Type 3 (62 carbons, A to N rings, DEF rings size 7/8/9). The structures shown are more energetically favourable (lower energy) epimers at carbon C52 for P-CTX-1 [98], P-CTX-3 [122], GT-4B [98], P-CTX-3C [123], at carbon C56 for C-CTX-1, C-CTX-2 [35], and C-CTX-5 [43]. The less energetically favoured epimers are shown in parentheses, while the unstable open (reduced) forms of the hemiketals C-CTX 3/4 [120] are not shown for clarity. Brevetoxin (PbTx-2) is shown for comparison [124]. A progressive numbering from 1 to 60 (Type 1), 1 to 57 (Type 2) or 1 to 62 (Type 3) was attributed for each contiguous carbon of the continuous chain of each type of CTXs and was completed at the end by numbering of methyl (Me); however, for clarity only some carbons were marked in rings of this figure: as an example in Type 1 it is pointed 5 for C5 of ring A; 25 for C25 of ring F; 52 for C52 of ring L.

More than 30 analogues with similar polyether backbones have been reported [19,74,118,119]. Representative backbone structures of the CTXs identified so far include those of P-CTX-1, P-CTX-3, GT-4B, CTX-3C, and C-CTX-1 (Figure 1 and Table 1). The main structural differences between C-CTXs and P-CTXs are localized at one end of the molecule, with a stable spiroketal (closed stable ring) compared to the hemiketal ring present in C-CTXs. Similar to sugars, the spiroketal and hemiacetal rings are in equilibrium, with an opened intermediate form allowing for interchange between higher and lower energy epimers/anomers, as seen in the well-known mutarotation in reducing sugars [120], with the lower energy form dominating. Importantly, this transition to the open form is acid-labile, and the levels of the open form are influenced by the conditions used during isolation [121].

Curiously, the C-CTX-1/-2 structure is closest to CTX-3C, with an identical size of successive rings from A to J. Overall, regardless of the family, different congeners of CTXs can be grouped into three backbone types (Figure 1, Table 1), two for P-CTXs and one for C-CTXs. Indian Ocean CTXs (I-CTX-1 to I-CTX-5) remain poorly documented [44,45,46,47], with their chemical structures, toxicity, and algal producer(s) still undetermined.

### 2.2. Availability of Ciguatoxin Reference Standards

The availability of different ciguatoxins is dependent on the amount that can be extracted from fish and/or produced by dinoflagellate cultures. However, the isolated amounts are extremely low, concentrations are in the ppb range, which is a major drawback for the development and validation of biological and analytical detection methods for CTXs. Indeed, the isolation and structural characterisation of pure toxins requires large amounts of highly toxic fish tissues collected in ciguatera endemic areas. In the 1990s, 125 kg of the viscera, including 43 kg of livers from four tons of moray eels (*Gymnothorax javanicus*), were collected in French Polynesia (Pacific Ocean) and extracted by Legrand et al. to obtain 350 µg of pure P-CTX-1 [127]. Lewis et al. extracted 48.3 kg of viscera from moray eels (*Lycodontis javanicus*) from the Republic of Kiribati (Pacific Ocean) to obtain 409 µg of P-CTX-1 [122]. Dickey et al. isolated 100 µg of pure C-CTX-1 from 164 kg of highly toxic specimens of barracuda and horse-eye jack [102], and Vernoux and Lewis isolated 150 µg of pure C-CTX-1 from 51 kg of liver, viscera, and flesh of horse-eye jack (*Caranx latus)* from Saint-Barthélemy (French West Indies) [38]. Currently, only the chemical structure of C-CTX-1 has been determined using nuclear magnetic resonance (NMR) data by Lewis et al. [25]. The structures of Pacific analogues have also been inferred using NMR and fast-atom bombardment mass spectrometry (FAB-MS) for analogues in low amounts [98,99,119,122,128].

The only CTX standards currently available commercially are: synthetic CTX-3C and P-CTX-1 from FUJIFILM Wako Chemicals Corporation; pure P-CTX-1, CTX-3C, CTX-3B, P-CTX-4A standards from the Institute Louis Malardé Phyconesia; and pure P-CTX-2 available from one of the authors (RJL). Recent production of NMR-calibrated standards of P-CTX-1, P-CTX-2 (or 52-epi-54-deoxyCTX1B), CTX-3C, 51-OH-CTX-3C, and P-CTX-4A from Japan [129] provide important, though incomplete, coverage of the ciguatoxins present in ciguateric fishes in the Pacific. Additionally, C-CTX-1 reference materials prepared from several fish from the northeast Atlantic Ocean [130] were recently characterised using capillary liquid chromatography–high-resolution mass spectrometry (LC-HRMS) [131]. Stable isotope standards of C-CTX-1/-2 using ^18^O labeling of the hydroxy group of the hemiketal at C56 and ^18^O-labeled C-CTX-3/-4 obtained through the reduction of C-CTX-1/-2 were produced by Mudge et al., in addition to two *Gambierdiscus* spp. metabolites: ^18^O-labeled gambierone and 44-methylgambierone [132]. These new reference standards provide useful analytical tools for the identification and quantification of C-CTXs using isotope dilution mass spectrometry.

## 3. Links between the Chemistry and Biochemistry and Toxicology of CTXs

### 3.1. Lipophilicity of CTXs

Known CTXs comprise 50 to 62 contiguous carbons, which are organized into 12–14 ether-fused rings that confer moderate lipophilicity and high chemical and thermal stability. The following facts have confirmed this first theoretical approach. Consistent with the chemistry, CTXs are soluble in aqueous acetone, acetonitrile, and methanol, but not in hexane, as originally determined by Hashimoto in the late 1960s [53,133]. This behavior is reminiscent of aflatoxins solubility [134,135]. Interestingly, in 1977, Scheuer [136] reported that the skin, fat, and bones of toxic moray *Gymnothorax funebris* caught in the Pacific Ocean were non-toxic. In a separate study, *G. funebris* specimens caught in the French West Indies (F.W.I) were shown to be ciguatoxic in flesh and other tissues, but not in fat (1 unit for flesh; ×7 for viscera, and ×45 for liver) [91]. This suggests that the ciguatoxins (P-CTXs and C-CTXs) found in *G. funebris*, which are not hexane-soluble, were not effectively soluble in the high fat content of *G. funebris* flesh and therefore did not accumulate in fats. The ciguatoxin concentration was the highest in the liver, which has a relatively low fat content compared to other tissues, either for Caribbean or Pacific fishes [36].

Lipophilicity has also been estimated from the partition coefficient of P-CTX-1 between ethyl acetate and a HEPES-buffered Ringer solution in a 1:1 ratio at 37 °C, which was >2, confirming that P-CTX-1 is a mid-lipophilic compound that can cross cellular membranes [137]. In line with this observation, P-CTXs were extracted from pooled mouse brains following IP (intraperitoneal) injection of LR, and from the brains of cats that were intoxicated after ingestion of ciguatoxic fish [62]. This finding correlates with the ability of CTXs to reach nerves and neurons to cause persistent, quasi-irreversible activation of voltage-gated sodium channels that increases neuronal excitability and neurotransmitter release, impairs synaptic vesicle recycling, and causes cell swelling [94,138,139,140,141,142]. Given their structural similarities, it is not surprising that the binding of CTXs overlaps with the brevetoxin binding site (site 5) on sodium channels [122,138,143,144].

The excitotoxicity and neurodegenerative effects of CTXs in mice can last several months after exposure to P-CTX-1 [145], as can the neurosensory [146] and neuropsychological effects, including depression, anxiety, and memory disturbances [2,147,148]. C-CTXs and P-CTXs have similar but sufficiently distinct structures and potency differences in mice that are within 10-fold less for Caribbean ciguatoxins. Molecular modelling has suggested that an asymmetry exists in binding within the sodium channels, but this finding requires experimental validation [149].

### 3.2. Distribution of C-CTXs in Fish

Caribbean CTXs distribute unevenly across fish tissues, including the liver, blood, kidney, spleen, skeletal muscle, heart, gonads, gills, skin, and bones [91,150]. This distribution among different tissues allows C-CTXs [151,152] and P-CTXs [89,153,154] to be retained in different amounts as they are transferred through the food chain. Their structural stability limits liver metabolism, which would normally be expected to accelerate depuration. Oxidation of P-CTXs enhances potency without appearing to dramatically accelerate depuration, given that the most oxidised form typically dominates in fish flesh [75,89]. Similar findings have been observed in *G. funebris* and other fish species, where the liver contains the highest concentration of C-CTXs. Various Caribbean fishes have been found to accumulate 5–43-times higher levels of C-CTXs in the liver than in flesh [91]. A study on 55 amberjacks from the Canary Islands, tested using the neuroblastoma cell-based assay (CBA-N2a), showed 17-times higher concentrations of C-CTXs in the liver compared to the flesh [112]. A recent study confirmed the unequal distribution of CTXs using CBA-N2a in fish by testing different muscle, liver, and gonad samples from four amberjacks (*Seriola* spp.) and four dusky groupers (*Epinephelus marginatus*) captured in the Canary Islands [155]. The caudal muscle exhibited more CTX activity than the other fractions of the fillet, while the highest ciguatoxin concentrations were found in the liver and gonads, from fish with lower levels of toxicity in their flesh [155]. Indeed, some fish that exhibited toxicity in liver may have had non-toxic and edible flesh [113]. This was also observed for Caribbean carnivorous fish (*Mycteroperca venenosa*, *Caranx bartholomaei*, *Seriola rivoliana*, *Gymnothorax funebris*) with edible flesh, despite their livers containing ciguatoxins at significant levels [156]. Not surprisingly, given its hydrophobicity, P-CTXs bind to proteins in fish liver [91] and skeletal muscle [157]. As fish nerves respond similarly to those of mammals, attachment to proteins could protect fish from the lethal effects of ciguatoxins [158]. This kind of protection is reminiscent of that observed for tetrodotoxin-contaminated invertebrates [159,160]. The amount of Pacific ciguatoxins has also been measured in fish blood [161], mouse blood [162,163], and human blood [164].

A study of fish liver and human P-450 enzymatic oxidation of P-CTXs by Ikehara et al. [90] confirmed that enzymatic oxidation of ciguatoxins occurs in the liver. Different rates of depuration, time from initial exposure, and species-specific differences in the ability of P-450 enzymes to generate C-CTX congeners can explain the different toxin profiles found in fish [19,23,38,41,42,45,46,75,89,91,118]. Two glucuronide products of C-CTX-1/-2 were recently identified by Gwinn et al. using high-resolution tandem mass spectrometry [165]. Their chemical reduction experiments confirmed that the metabolites were comprised of four distinct glucuronide products, with the sugar attached at two separate sites on C-CTX-1/-2, and the hydroxyl group on carbon C56 was excluded as the conjugation site [165]. Glucuronidation is a novel biotransformation pathway that has not been reported for other related polyether phycotoxins, yet its occurrence across all the tested fish species suggests that it could be a prevalent and important detoxification mechanism in marine organisms. The absence of glucuronidation observed in rat and human microsomes suggests that alternate biotransformation pathways may be dominant in higher vertebrates [165].

### 3.3. Stability of CTXs

The mouse bioassay has determined that P-CTXs levels are not affected by freezing, cooking at 120 °C in water, drying, or salting [166]. When diluted in olive oil, 100% of the toxicity was retained at <200 °C [94]. Furthermore, in water, pyridine and acetic acid P-CTXs solutions were stable at 100 °C for at least 10 min [167]. Few studies have evaluated the stability of CTXs in various temperature or acid/basic conditions. Vernoux and Bagnis [59] confirmed that rapid cold alkali treatment for P-CTXs, which was used to facilitate rapid defatting of extracts, did not affect the toxicity in mice or the preparative thin layer chromatographic behaviour. Alkaline stability was later confirmed using purified P-CTX-1 [94,167]. However, ~50% of C-CTXs were lost upon alkaline treatment, making this rapid defatting step unsuitable for C-CTX extraction or quantitation. Based on the organic solvents used during purification, P-CTXs and C-CTXs are stable in acetone, diethyl ether, chloroform, benzene, and ethanol, with methanol preserving ciguatoxins for months [167] and most stable forms for years (Vernoux J.-P. and Lewis R.J., unpublished results). C-CTX-1 remained unchanged after six months of storage at −10 °C in 50% aqueous acetonitrile, or after 4 h in 50% acetonitrile/5% trifluoracetic acid [35]. However, C-CTX-2 was converted into C-CTX-1 after six months of storage at −10 °C in 50% aqueous acetonitrile [35]. In an acidic medium, (1 M HCl) at ambient temperatures, C-CTXs were stable [94], while the activity of P-CTXs decreased by >50% at room temperature and was completely lost at 100 °C [167]. Talha [168] assayed C-CTXs with specific reagents and reported a total disappearance of activity when targeting -OH or a vicinal diol moiety with HIO_4_ in acetic acid for 2 h at 25 °C, or with acetic anhydride in pyridine 1 h at 100 °C. A similar loss of toxicity was observed when targeting double bonds between two adjacent carbons, with the addition of OsO_4_ for 3 h at 25 °C, the addition of the oxidants KMnO_4_ for 30 min at 25 °C, or H_2_O_2_ into CH_3_COOH for 2 h at 85 °C, while the activity of C-CTXs in the controls remained unaffected [168]. Thus, these experiments suggest that primary or secondary hydroxyl(s) and carbon double bonds are major structural determinants of C-CTX activity. These key features are consistent with the structures of the major CTXs found in fish [35,98,99,125]. The instability of C-CTX-1 in strongly acidic conditions was recently demonstrated using methanol (or acetonitrile) and a C56 methoxy congener, C-CTX-1-Me, which was structurally characterized using its mass fragmentation pathways in liquid chromatography-tandem mass spectrometry and high-resolution mass spectrometry [121,131]. Thus, using strongly acidic conditions during sample pre-treatment for C-CTX analysis is expected to adversely affect the ability to quantitatively determine the levels of C-CTX-1 in extracts of fish or dinoflagellates [121].

## 4. Extraction and Purification of C-CTXs

As fish ciguatoxins are moderately lipophilic and found as trace contaminants, this ppb range (and below) poses a serious challenge for chemical detection. Thus, the removal of co-extracted fats and liposoluble contaminants is an important step for the preparation of reference/pure toxin standards. The purification must efficiently extract the toxins and minimize matrix effects by eliminating interfering compounds in order to improve detection using bioassays and MS methods [169]. The protocols used for ciguatoxin extraction from flesh and purification for LC-MS analysis are multistage procedures that typically include acetone extraction, liquid–liquid partitioning between diethyl ether and water, and defatting using hexane. The resulting crude extract requires additional solid phase extraction (SPE) clean-up on normal-phase and/or reversed-phase adsorbents before analysis (for review, including P-CTXs extraction and purification methods, see [118,169,170]).

### 4.1. Extraction and Purification Methods for C-CTXs Isolation (>1 kg of Fish Flesh)

It is important to note that the extractive power of acetone is enhanced by the initial presence of water in flesh, and it is less efficient on dry flesh. Raw or cooked fish tissue can be used, as toxin profiles appear to be unchanged by cooking, which matches the cooked flesh typically consumed [23,91,104,108]. Thus, after an initial extraction of wet flesh using acetone, a second extraction must be performed using acetone/water 80:20 (*v*/*v*) to achieve >90% recovered toxicity [38], and a third acetone extraction is unnecessary [91]. It is better to perform a single extraction, followed by rinsing the cake with 80% aqueous acetone, which has the advantage of significantly reducing the contaminant/ciguatoxin ratio [104]. Both suction chromatography and flash chromatography were shown to save time when using 5–25 kg of fish tissue [104,167]. A summary of the extraction and purification methods for isolation of C-CTXs from large quantities of Caribbean fish (several kg) has been published in [38,104].

**Figure 2:**
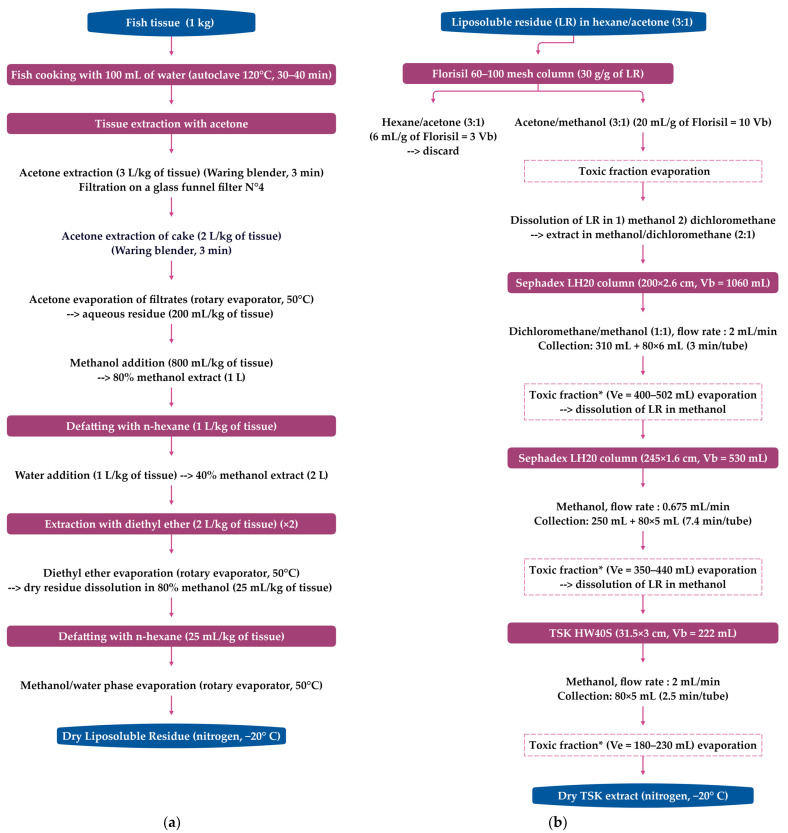
Flow diagram of the C-CTX extraction and purification stages used to study Caribbean fish toxin profiles using LC-MS-radiolabeled-ligand binding [23,40,42] and to isolate C-CTX-1 and -2 [35,38]. (**a**) Extraction stage, (**b**) purification stage. * Toxicity was assessed using the mouse bioassay (MBA) on the fractions bordering toxic peaks. The fractions were evaporated under nitrogen, and 1/100 or 1/50 of each fraction was dissolved in 0.6 mL of 1% Tween 60 for IP (intraperitoneal) injection in mice. Vb = bed volume, Ve = elution volume.

**Figure 3:**
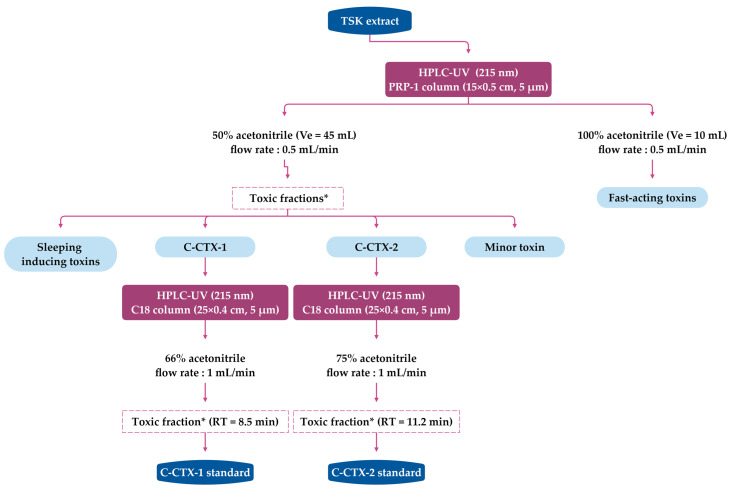
Flow diagram of C-CTX purification using HPLC-UV for the isolation and characterisation of C-CTX-1 and -2 standards from TSK (Toyopearl^®^ HW 40S) extracts of horse-eye jack tissues [35,38]. * Fractions were evaporated under nitrogen, and up to 10% of the collected fractions was dissolved in 0.6 mL 1% Tween 60 for IP injection in mice (MBA). Ve: elution volume, RT: retention time.

A flow diagram of the isolation and purification of fish tissue used to study toxin profile in Caribbean fish [23,40,42] is provided in Figure 2. This protocol was adapted from the procedure developed for P-CTXs by Lewis et al. [122,171], with the first preparative chromatography on the silica phase replaced with Florisil^®^ to avoid acid catalysed spiroisomerisation of spirochaetal on silica gels [1]. This approach allowed for the isolation and structural characterisation of C-CTX-1 and its epimer C-CTX-2 from horse-eye jacks (*C. latus*) [35,38]. The TSK (Toyopearl^®^ HW 40S)-purified extracts from *C. latus* were then subjected to reversed-phase HPLC-UV (Figure 3) to isolate 90% pure C-CTX-1, C-CTX-2, and three other toxic fractions (using MBA) that accounted for <20% of the total toxicity: a sleep-inducing fraction (<1% of the total toxicity), a minor toxin (~1% of the total toxicity), and a fraction known as the fast-acting fraction (19% of the total toxicity) [35,38]. At the time of these publications, their relationship to fish ciguatoxins had not been determined, but the lack of penile symptoms in mice indicates that their mode of action in vivo was likely different from the known ciguatoxins.

From a highly toxic amberjack extract, Poli et al. also used reverse chromatography to separate two minor fractions in addition to a fraction containing C-CTX-1 [172]. These two minor fractions, one more polar than C-CTX-1 and the other less polar, were active in the RLB assay [172]. Other fast-acting toxins with effects identical to CTXs but much more pronounced were later found in coarser extracts [41,104] (see Section 5.1.2).

Another protocol, adapted from the Dickey R.W. procedure (unpublished data) [102], was recently used to prepare C-CTX-1 reference materials from fish tissue (50 kg) and fish liver (6 kg) from the Canary Islands (Spain) and the Madeira archipelago (Portugal) [130]. The main differences from the previous protocol (Figure 2) lie in the sequencing of the extractions and the use of SPE cartridges (Florisil^®^ and C8) for chromatographic purification. Tudó et al. compared the order of the liquid–liquid extraction (LLE) procedures and showed that when the diethyl ether extraction is performed prior to hexane cleaning, detection of C-CTX-like toxicity using CBA-N2a can be enhanced [113]. However, it remains to be determined if this change affects LC-MS detection.

To study the diversity of ciguatoxins and control the presence of toxins in fish implicated in CP, the extracted lipid-soluble residue can be submitted to additional purification procedures to remove additional non-polar and polar compounds using solid phase extraction (SPE) on silica [39,107,108,120], amino [173], aminopropyl [37,174], or Florisil^®^ [109,110,112,175,176] cartridges. In some studies, a C18 reversed-phase SPE clean-up step is added to further reduce matrix effects. After the last CTX-containing eluate is evaporated to dryness, the residue is dissolved in methanol and filtered prior to LC-MS analysis.

### 4.2. Rapid Extraction Methods for CTXs (<5 g of Fish Flesh)

**Figure 4:**
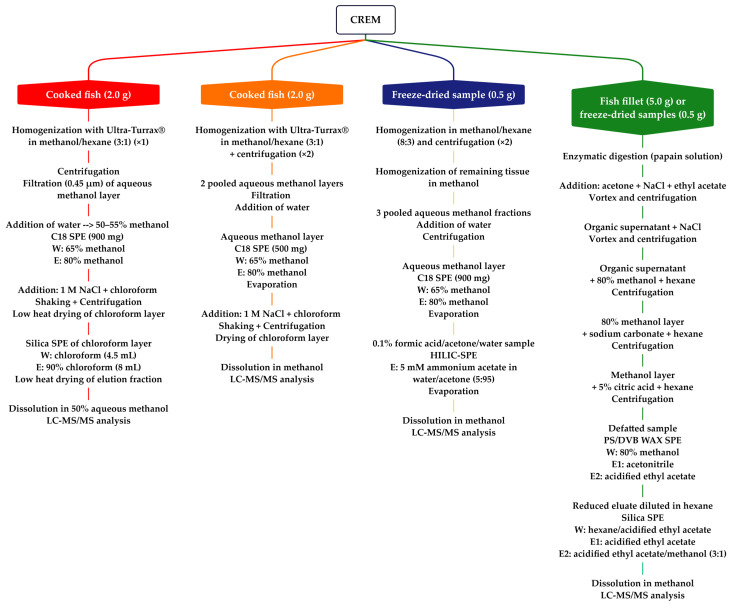
Comparison of flow diagrams of ciguatoxin rapid extraction methods (CREM) developed by Lewis et al. [177] (red) and further modified by Stewart et al. [178] (in orange) and Meyer et al. [179] (in blue), and an alternative approach proposed by Spielmeyer et al. [180] (in green).

To improve the efficiency of ciguatoxin extraction and HPLC-MS/MS detection, ciguatoxin rapid extraction methods (CREM) sampling small amounts of flesh (<5.0 g), have been applied to P-CTXs analysis [177,178,179,180] (Figure 4). Lewis et al. [177] developed the first CREM that used a novel approach to CTX extraction, and the clean-up and was optimized to:

1. Reduce the quantity of fish flesh extracted (≤2 g).
2. Replace the initial acetone extraction and filtration step with a one-step extraction and hexane cleanup.
3. Reduce the number transfer and drying steps.
4. Use centrifugation to speed the separation of phases.
5. Consolidate LLE to a single step.
6. Incorporate two orthogonal SPE cleanup steps on C18 and silica.

CREM allowed for the quantification of P-CTX-1 in Australian fish at levels of ≥ 0.1 ppb [177] and the screening of Pacific and Caribbean isolates of *Gambierdiscus* for MTX-like and CTX-like activity [81]. Stewart et al. then successfully adapted the CREM approach in an analytical laboratory setting for routine screening of suspect ciguateric fishes in Queensland (Australia) using P-CTXs standards supplied by Lewis [178]. Meyer et al. later introduced a hydrophilic interaction liquid chromatography (HILIC) SPE cleanup instead of the C18 SPE cleanup, which improved P-CTX-1, -2, and -3 extraction efficiency but required an additional 85 min per batch of 12 samples [179]. To our knowledge, none of these CREM-LC-MS/MS methods have been used to detect and quantify clinically relevant levels of C-CTXs in fish flesh from the Atlantic. However, given their chemical similarities, these approaches are expected to be useful for the analysis of fish containing C-CTXs.

In 2021, Spielmeyer et al. developed and partially validated a new extraction protocol for multiple CTXs from all regions by introducing an enzymatic digestion for the first stage, followed by acetone extraction, defatting with modified n-hexane (with either sodium carbonate or 5% citric acid), a polar PS/DVB WAX SPE (polystyrene-divinylbenzene copolymer with a weak anion exchanger) and normal-phase SPE on silica [180]. However, due to the lack of reference material for C-CTX-1 and the absence of any standard for I-CTX-1, the recovery rates (35–88%) and matrix effects (66–116%) were determined using LC-MS/MS for only P-CTX-1, P-CTX-2, P-CTX-3, and P-CTX-3C. This method was applied to fish presumed to have been caught from the southwest coast of India, but with CTX profiles identical to those of fish from the Pacific Ocean (see Section 5.2.4). Additionally, this protocol [180] needs to be validated on Atlantic fish to ensure that the use of acidified solutions is not detrimental to C-CTXs extraction efficiency.

Wu et al. also validated a rapid extraction method using accelerated solvent extraction (ASE) for the detection and quantification of P-CTX-1 in fish flesh (5.0 g) using HPLC-MS/MS. Using a sensitive MS system (5500 QTRAP, AB Sciex, Foster City, CA, USA), the validated method had a limit of quantitation (LOQ) of 0.05 ppb in fish muscle and matrix spike recoveries ranging from 49% to 85% in 17 coral reef fish species [181].

## 5. Analytical Methods for Detection, Identification, and Quantification of C-CTXs

Currently, there is no official method for CTX monitoring of food in Europe, although it is stated in European regulation that “Controls shall take place to ensure that fishery products containing biotoxins such as ciguatera or other toxins dangerous to human health are not placed on the market” (Commission Implementing Regulation (EU) 2019/627). In the Pacific, Lehane and Lewis [16,182,183] noted that mild ciguatera outbreaks occur after exposures to 0.001 ppb of P-CTX-1. Based on these estimates, a 10× risk factor was applied to estimate that the concentration of a ‘safe’ fish would contain no more than approximately 0.01 ppb P-CTX-1 [16]. The U. S. Food and Drug Administration set a guidance value 10-fold below the lowest observed adverse effect level (LOAEL) determined by Lewis et al. [16], which was 0.01 ppb P-CTX-1 equivalent [184]. This concentration is just below the lowest concentration (0.022 ppb P-CTX1 eq.) seen in fish samples associated with CP cases in Guadeloupe [22]. However, in this article [22], the ciguatoxin content was evaluated using P-CTX-1 as reference material. Based on the ratio of LD_50_ in IP injected mice between C-CTX-1 (LD_50_ = 3.6 µg/kg [38]) and P-CTX-1 (LD_50_ = 0.25 µg/kg [122,171]), a guidance level of 0.1 ppb C-CTX-1 equivalent was proposed for C-CTX-1 [7,184]. In 2010, the CONTAM Panel of the European Food Safety Authority (EFSA) also indicated that a concentration of 0.01 ppb P-CTX-1 eq. in fish is not expected to exert effects in sensitive individuals eating a significant amount of contaminated fish [185], although maximum CTX limits for fish still have not been established for regulatory purposes in Europe [26].

Ciguatoxin detection is a significant analytical challenge due to the low concentrations of toxins in complex matrices such as fish flesh and the diversity of toxin profiles encountered in ciguateric fishes. The methodological approach for ciguatoxin monitoring usually involves a two-tier approach, with the first tier using a bioassay screen and the second tier using analytical methods. Screening can be carried out on fish extract or during chromatographic fractionation using the mouse bioassay (MBA), which has been used for decades [23,38,42,156,166,171]. For example, Hoffman et al. [186] used female mice, and Vernoux [166] used male mice to specifically allow for the observation of characteristic penile symptoms associated with all CTXs, regardless of their geographic origin [19]. Alternatives to in vivo assays include cytotoxicity assays [37,39,108,112,143,170,174,175,176,187,188,189], calcium influx assays [81], radio-ligand binding assays (RLB), currently known as competitive sodium channel receptor binding assays (RBA) with radiolabeled brevetoxin-3 (rRBA) [23,41,45,144,172,190,191], or recently developed fluorescence (fRBA) [173,192] and chemiluminescence assays [193]. In the case of Caribbean ciguatoxin analysis, improved sensitivity was reported for cell-based assays (CBA) over RBAs [173,194]. In the second tier, analytical methods typically employ reversed-phase liquid chromatography (LC) coupled to electrospray (ESI) mass spectrometry (MS) detection. These methods were initially developed for Pacific CTXs [32,33,171], and later for Caribbean CTXs [23,38,41,42,172]. They can be used to confirm the presence of known CTXs and CTX-like compounds in fish and dinoflagellate extracts and to quantify their levels when analytical standards are available. A number of published reviews have summarised the advantages and disadvantages of bioassays and analytical methods [6,19,96,114,118,170,195,196,197,198].

### 5.1. Analysis of C-CTXs in Caribbean Fish Using HPLC-MS (Low-Resolution)

#### 5.1.1. First Characterisation of C-CTX-1 and C-CTX-2 in Caribbean Fish

The first mass spectrum of C-CTX-1 acquired using an ESI-triple quadrupole mass spectrometer in 1995 suggested that C-CTX-1 has a molecular ion at *m*/*z* 1123 [102]. A triple quadrupole mass spectrometer was also used to confirm the presence of an ion at *m*/*z* 1123 in partially purified extract of the greater amberjack *Seriola dumerili*, which was implicated in an outbreak of ciguatera among US soldiers in Haiti [172]. However, subsequent MS analysis revealed this ion was associated with the first loss of water from the [M+H]^+^ ion at *m*/*z* 1141 [38], which was later confirmed when the full structure of C-CTX-1 was elucidated using NMR [35]. Moreover, in the same study, two minor toxins that targeted receptor site 5 on the voltage-dependent sodium channel, one more polar and one less polar than C-CTX-1, were also found through HPLC-UV analysis [35]. 

The first characterisation using HPLC-MS with water/acetonitrile eluents modified with 0.1% trifluoroacetic acid (TFA) and an ionspray-triple quadrupole (PE-Sciex API III) was performed on pure Caribbean C-CTX-1 and -2 extracted from 51 kg of horse-eye jack (see Figure 2 and Figure 3) [38]. This low energy MS approach revealed a series of positive ions corresponding to the pseudo-molecular ion [M+H]^+^ at *m*/*z* 1141.6 and five successive water losses [M+H–nH_2_O]^+^, as well as potassium [M+K]^+^, sodium [M+Na]^+^, and ammonium [M+NH_4_]^+^ adducts associated with the parent ion [38]. HPLC-MS analysis of this extract revealed the presence of up to five C-CTXs, including C-CTX-1, C-CTX-2, and two coeluting compounds that were less hydrophobic than C-CTX-1. These compounds were assigned [M+H]^+^ ions of *m*/*z* 1157.6 and *m*/*z* 1143.6 Da, respectively. Another compound with a presumed [M+H]^+^ ion of *m*/*z* 1107.6 Da was observed, but its relationship to C-CTX-1 remains unclear [38]. Indeed, during the purification of the lipid-soluble residue obtained from pooled horse-eye jacks, several fractions were toxic in mice [35,38], as indicated in Figure 3 (see Section 4.1).

#### 5.1.2. Combined Analysis of Toxin Profiles in Caribbean Fish Specimens (Horse-Eye Jacks, Grey Snapper, Grouper, Black Jack, and Barracuda) Using MBA, HPLC-MS, and RLB

Based on their retention time and typical MS fragmentation compared to the C-CTX reference standards [35,38], C-CTX-1 and C-CTX-2 were identified and quantified in flesh extracts (see Section 4.1 and Figure 2) of horse-eye jack from Saint-Barthélemy (F.W.I) [42]. Using a water/acetonitrile gradient, the detection of C-CTX-1 characteristic ions (adducts and water losses) and the signal/noise ratio were improved using formic acid-modified eluents instead of trifluoracetic acid-modified eluents [42]. Formic acid-modified eluents enhanced the area of the base peak chromatogram by 2.7-fold and the peak height by 3.4-fold when the major ion [M+H–H_2_O]^+^ was extracted [42]. Individual analysis of fish profiles using MBA [166] and HPLC-MS showed that, contrary to low-toxic *C. latus* specimens (75–90% of C-CTX-1), highly ciguatoxic fish had C-CTX-1 levels < 50% that varied independently of mouse toxicity and were subjected to large losses of activity during purification, suggesting unstable ciguatoxins were also present [40,42]. A search for CTX with characteristic patterns of ion formation in MS (for details, see Table 2 in [42]) revealed five C-CTX congeners with pseudo-molecular ions at *m*/*z* 1141.58, 1143.60, 1157.57, 1159.58, and 1127.57, and additional isomers at *m*/*z* 1141.58 (2 isomers), 1143.60 (1 isomer), and 1157.57 (2 isomers) were present only in some flesh extracts [42]. A nomenclature based on [M+H] ions was defined to identify these new C-CTX analogues, and a hypothetical structural assessment was proposed for C-CTX-1143 and its isomers (putative reduced forms of C-CTX-1/-2, later named C-CTX-3/-4 in [120]), C-CTX-1157 and its isomers (putative oxidized forms of C-CTX-1/-2, later named 17-hydroxy-C-CTX-1 in [131]), C-CTX-1159 (possible addition of water to a C-CTX-1 double bond), and C-CTX-1127 (possible loss of a -CH_2_ group or loss of a methyl group from C-CTX-1). A comparative analysis of raw and cooked flesh in two specimens showed that cooking did not modify toxicity in mice and that the toxin profiles were identical, with the relative proportion of each C-CTX class insignificantly changed by cooking [42]. Any conversion of C-CTX-1 into its closely eluting isomer C-CTX-1141a after cooking did not change toxicity, suggesting that both CTXs had similar toxicity [42]. Given the lack of standards for most C-CTX congeners, their relative contribution to toxicity was assessed by grouping the LC-MS peak areas of C-CTXs of the same mass, as a function of the toxin concentration in the flesh determined using the mouse bioassay for 12 *C. latus* specimens. These included C-CTX-1 (4 isomers), C-CTX-1143 (2 isomers), C-CTX-1157 (3 isomers), and C-CTX-1127. While no quantitative correlation with flesh toxicity was found, the C-CTX-1157 isomers increased from ~2 to 20% with increasing fish toxicity [42].

**Figure 5:**
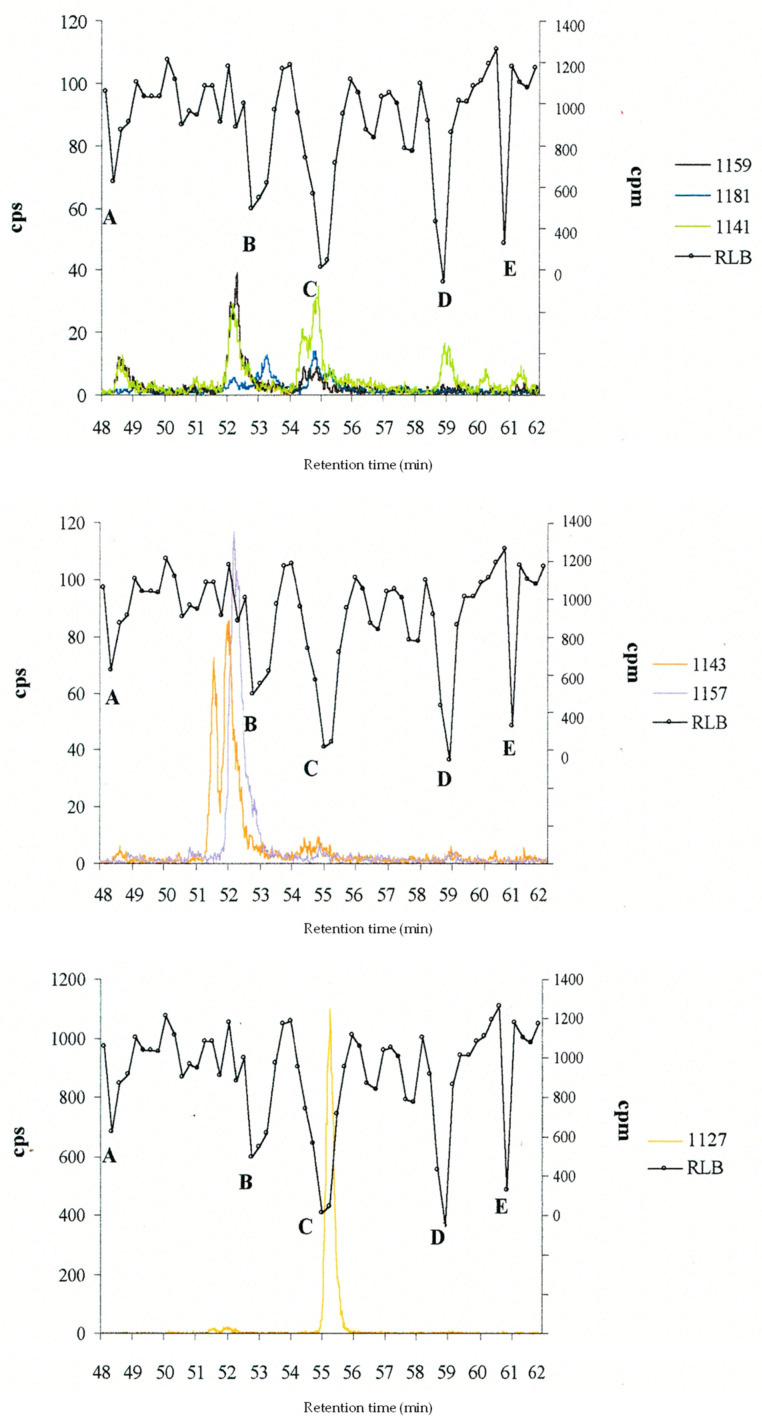
Extracted chromatograms (in cps) for C-CTX-1143 (referred to as C-CTX-3/-4 in [120]), C-CTX-1157, C-CTX-1127, C-CTX-1159, C-CTX-1181, and C-CTX-1141 from HPLC-MS [42] and radiolabeled-ligand binding (RLB in cpm) analysis (unpublished data from [40]) of a TSK extract from a Caribbean horse-eye jack (*C. latus*) known as CL5 in [42]. Peaks A to E correspond to binding of CTX analogues to Na^+^ channels. LC-MS analyses were conducted using a Zorbax C3 300 SB (2.1 × 150 mm) column with a water/acetonitrile gradient containing 0.1% formic acid and a Quadrupole Time-of-Flight PE Sciex QStar Pulsar.

Subsequently, an extract of an inedible horse-eye jack, known as CL5 [42], was analysed using HPLC-MS and radiolabeled-ligand binding assay (RLB) simultaneously (unpublished data from [40]). This additional combined analysis revealed five peaks of binding to sodium channels (A to E) (Figure 5 and Table 2). Unfortunately, some peaks co-eluted (peaks A, B and C), which did not allow for sodium channel affinity to be assigned to specific congeners (Table 2). Moreover, in the toxic fraction A containing C-CTX-1159, two other pseudomolecular ions with unusual masses for CTX were detected: [M+H]^+^ at *m*/*z* 859.42 with sodium and potassium adducts and four water losses, and [M+H]^+^ at *m*/*z* 811.47 with only four water losses (Table 2).

The putative C-CTX analogues identified in the horse-eye jacks study [42] were also identified in other fish specimens implicated in CP in Guadeloupe (F.W.I.), including a grey snapper (*Lutjanus griseus*), an unidentified grouper (*Serranidae* sp.), and a black jack (*Caranx lugubris*) [23]. Specifically, C-CTX-1 was quantified using pure C-CTX-1 standard, at 0.24, 0.90, and 13.8 ppb in the snapper, the grouper, and the black jack, respectively. However, this only partially contributed to the overall toxicity determined using the MBA [23]. Other identified toxins were C-CTX-2, three additional isomers of C-CTX-1 or -2, and five C-CTX congeners (C-CTX-1127, C-CTX-1143 and its isomer C-CTX-1143a, and C-CTX-1157 and its isomer C-CTX-1157b). Putative hydroxy-polyether-like compounds were also detected in the grouper flesh, with [M+H]^+^ ions at *m*/*z* 851.51, 857.50, 875.51, 875.49, and 895.54 Da [23]. Further HPLC-MS and RLB analyses of the TSK-purified extract of black jack (*C. lugubris*) identified C-CTX-1 and C-CTX-1127. However, due to their close elution, it was not possible to confirm whether C-CTX-1127 has an affinity for sodium channels (unpublished data from [40], pp. 197–198).

In one highly ciguatoxic specimen of barracuda (*Sphyraena barracuda*), as determined by MBA [41], two distinct fractions (FrA and FrB) were separated during the first fractionation stage on Sephadex LH20 (gel filtration chromatography), resulting in two different response profiles in IP injected mice, as previously observed with *S. barrucuda* and *G. funebris* [104]. It should be noticed that the Florisil^®^ chromatography (Figure 2) was bypassed because barracuda ciguatoxins have previously shown a lack of stability during this purification stage [104]. The main toxic fraction, FrA, produced a delayed onset of symptoms in IP injected mice, typical of slow-acting toxins (diarrhea, dyspnea, penile cyanosis, hypersalivation, shortest survival time of ~30 min). The second unknown toxic fraction, FrB, induced a rapid onset of symptoms, typical of fast-acting toxins (irritability, hypersalivation, dyspnea, penile cyanosis, tail arching, hindquarters paralysis, ataxia of forelimbs, shortest survival time of ~10 min). HPLC-MS analysis confirmed that FrA and FrB were distinct, with only a weak overlap of some compounds. C-CTX-1 accounted for 90% of the total toxicity of the barracuda flesh (49 ppb in FrA). C-CTX-2, C-CTX-1141b, C-CTX-1143, and C-CTX-1157a (traces) were also identified, but they did not show any significant activity in the RLB assay. Conversely, fast-acting toxins active on voltage-gated sodium channels with molecular masses <900 Da were suspected to contribute to the overall toxicity. Indeed, despite important losses in toxicity during further purification of FrB (>60%), peak sodium channel activity was identified, and two pseudo-molecular ions at *m*/*z* 809.43 and *m*/*z* 857.42 were detected using LC-MS in the corresponding fraction, suggesting that one or both may account for the FAT effects in mice [40,41]. The capacity of fish to detoxify ciguatoxins was already shown for *S. barracuda* by Tosteson et al. [199]; therefore, we hypothesized that these fast-acting toxins could be metabolites of C-CTXs or of other toxins with a molecular mass within the same range as the brevetoxins [41].

In conclusion, the cited HPLC-MS studies carried out in the early 2000s on the toxin profiles of different species of fish from Saint Barthélemy and Guadeloupe were the first to highlight the multiplicity of ciguatoxins in the Caribbean. These included C-CTX-1 and -2 (confirmed with standards), C-CTX-1141 (3 isomers), C-CTX-1159, C-CTX-1143 (2 isomers), C-CTX-1157 (3 isomers), C-CTX-1181, and C-CTX-1127 [23,38,41,42]. In addition to C-CTX-1 and -2, the presence of sodium channel activator toxins was also revealed in one horse-eye jack specimen and one highly toxic barracuda. However, challenges separating minor from major CTX did not allow for a reliable link to be demonstrated between the toxic fractions in the radiolabeled-ligand binding assay and the chromatographic peak of a specific CTX [23,41,42]. However, this correlated with a previous study using improved LC-MS/MS for the determination of CTXs at sub-ppb levels in crude fish extracts (8 toxic, 12 borderline, 10 non-toxic using MBA), which estimated that ~50% of the toxicity determined using the MBA was due to the presence of C-CTX-1 [200].

### 5.2. Analysis of C-CTXs in Fish by HPLC-MS/MS (Low-Resolution)

#### 5.2.1. First Quantification of C-CTX-1 in Caribbean Fish Using HPLC-MS/MS

A significant advance in terms of ciguatoxin detection was achieved with the arrival of HPLC coupled with tandem mass spectrometry (HPLC-MS/MS). Using pure standards of P-CTX-1 and C-CTX-1 (and PbTx-2), HPLC-MS/MS signals for the detection of P-CTX-1 and C-CTX-1 were acquired using an ionspray-triple quadrupole (PE-Sciex API III) coupled to a HPLC eluted using a water/acetonitrile gradient modified with 0.05% TFA [200]. Fish samples from Saint Barthélemy (F.W.I.) were also tested using MBA and analysed in the positive ion mode. The MS/MS signals were optimized for ions originating either from the loss of NH_3_ or the additional losses of one, two, or three H_2_O molecules from the dominant [M+NH_4_]^+^ ion [200]. Despite negative matrix effects of 37% for C-CTX-1 and 15% for P-CTX-1, this method allowed their detection at clinically relevant levels in fish flesh, with detection limits of 0.1 ppb and 0.04 ppb for C-CTX-1 and P-CTX-1, respectively, in 2.5 g of fish flesh equivalent injected in LC [200]. This first study on several Caribbean fish of variable toxicity (non-toxic, borderline, toxic by the MBA) individually extracted from 50 g of fish flesh showed that, considering a maximum safe level of C-CTX-1 set at >0.25 ppb, 5 of 12 borderline toxic fish and all toxic and inedible fish contained levels of C-CTX-1 that were likely to affect humans. Moreover, ~50% of the toxicity determined using the MBA was due to the presence of C-CTX-1 [200].

#### 5.2.2. Identification and Quantification of C-CTX-1 in Fish from Macaronesia Using HPLC-MS/MS with Water/Methanol LC Gradients and SRM on Precursor Ions [M+Na]^+^

Using an LC mobile phase of methanol modified with ammonium formate and formic acid to produce high-intensity signals for stable [M+Na]^+^ ions on ESI-triple quadrupole, Yogi et al. [30] monitored the sodium adducts of known Pacific CTXs as precursor ions in Q1 and as product ions in Q3. These LC conditions allowed for single reaction monitoring (SRM) of [M+Na]^+^ > [M+Na]^+^ transition for each CTX, and with high collision energy, it was possible to eliminate background noise and monitor the sodium product ions without any additional fragmentation [109]. This targeted MS analysis was applied to quantify C-CTX-1 in several fish species along the North-East Atlantic coasts as part of the European project “EuroCigua: Risk Characterization of Ciguatera Fish Poisoning in Europe” (Table 3) [109,110,112,155,175,176].

Using this method, C-CTX-1 was detected using LC-MS/MS in eight out of eleven samples from the Selvagens Islands, but only three fish had C-CTX-1 levels above the LOQ: a 19-kg dusky grouper, *Epinephelus marginatus* (0.05 ppb); a 1.6-kg Barred Hogfish, *Bodianus scrofa* (0.11 ppb); and a 4.5-kg island grouper, *Mycteroperca fusca* (0.25 ppb) [109]. No CTXs were detected in any of the fish samples from Madeira Island [109]. C-CTX-1 levels above the FDA guidance levels of 0.1 ppb C-CTX-1 eq. were also quantified using HPLC-MS/MS in four fish implicated in CP and with a positive CTX-like toxicity (by CBA-N2a): a dusky grouper, *Epinephelus marginatus* (0.12 ppb); an amberjack, *Seriola* spp. (0.37 ppb); a cubera snapper, *Lutjanus cyanopterus* (0.49 ppb) from the Canary Islands (Spain); and a red Porgy, *Pagrus Pagrus* (0.76 ppb) from the Selvagens Islands (Portugal) [110]. In a 37-kg amberjack, *Seriola fasciata*, implicated in a CP and captured near the Selvagens Islands (Portugal), the C-CTX-1 level was 0.84 ppb C-CTX-1 eq. using LC-MS/MS, whereas its composite toxicity was evaluated at 1.4 ppb C-CTX-1 eq. using the CBA-N2a [175]. Costa et al. reported that C-CTX-1 was the only CTX analogue identified using LC-MS/MS in all the species evaluated, with concentrations of C-CTX-1 of up to 0.48 ppb in barred hogfish (*B. scrofa*), a species with an intermediate position in the food web [176]. Finally, when Ramos-Sosa et al. compared the CTX content of the flesh and liver of 109 specimens from the Canary Islands (Spain) [112], CTX-like toxicity was detected using CBA-N2a in 107 livers out of 109. A total of 93 of the fish (85.3%) also showed toxicity in their flesh, with large differences in CTX concentrations found between tissues of the same fish, in particular in black moray eel (*Muraena helena*) specimens [112]. Of the 62 fish specimens further analysed using LC-MS/MS, the presence of C-CTX-1 in flesh was confirmed for only 30 fish samples at levels ranging from 0.018 to 0.270 ppb of C-CTX-1. C-CTX1 was the only CTX analogue identified among the monitored CTXs [112].

In all these studies on fish from the East Atlantic Ocean [109,110,112,155,175,176], no identified Pacific CTXs were detected, and discrepancies between toxicity determined using CBA-N2a and C-CTX-1 levels quantified using LC-MS/MS, although this difference was not evaluated for statistical significance. This may suggest the presence of other toxic analogues in the fish from this area and/or a toxicity overestimation arising from the cell-based assays and/or that matrix suppression was not adequately assessed across species.

#### 5.2.3. Identification of C-CTX-1 and Identification of C-CTX Congeners in Fish Using HPLC-MS/MS with Water/Acetonitrile LC Gradients

Lewis and Jones [33] first demonstrated that acetonitrile–water gradients buffered with 1 mM of ammonium acetate improved the separation and detection of Pacific ciguatoxins using HPLC-MS compared to a water/acetonitrile gradient modified with 0.1% trifluoroacetic acid (TFA). Subsequently, acetonitrile–water gradients modified with formic acid, ammonium acetate, or ammonium formate were used to enhance the detection of the major ion [M+H–H_2_O]^+^ of C-CTX-1 in LC-MS [42] and LC-MS/MS [37,39,108,174]. The LC-MS/MS methods that have been developed since the 2010s either on ESI-quadrupole-linear ion trap instrument [37,39,108,174] or ESI-triple quadrupole [110,175,176] confirmed C-CTX-1 presence in fish and tentatively identified new C-CTXs congeners. This approach also allowed for links between ciguatoxins prevalence and regional fish species distribution to be established in the Caribbean Sea and in the Eastern Atlantic Ocean, as detailed below.

- Identification of C-CTX-1 and C-CTXs congeners in Caribbean Fish

The (U)HPLC-MS/MS methods developed using an ESI-quadrupole-linear ion trap instrument (QTRAP 4000, Applied Biosystems, Waltham, MA, USA) confirmed the presence of C-CTX-1 in Caribbean fish that tested positive for CTX in the neuroblastoma cell-based assay (CBA-N2a). This conformation was achieved by comparing the retention time and ion transitions relative to a purified C-CTX-1 standard [22,37,39,108,174] (Table 4).

Based on extracted samples of a barracuda implicated in a CP and purchased in a local supermarket in Massachusetts (USA), Abraham et al. evaluated composite toxicities of 1.6 and 2.1 ppb in the cooked meal remnant and uncooked portion, respectively [108]. LC-MS/MS analyses confirmed the presence of C-CTX-1 and identified additional putative C-CTX congeners. In this work [108], the in-source collision-induced dissociation (CID) of C-CTX-1 [M+H]^+^ *m*/*z* 1141.6 resulted in the loss of water and a major fragment ion at *m*/*z* 1123.6 [M+H–H_2_O]^+^. Therefore, three precursor/product ion transitions were selected for multiple reaction monitoring: *m*/*z* 1123.6 → *m*/*z* 1105.6, *m*/*z* 1123.6 → *m*/*z* 1087.6, and *m*/*z* 1123.6 → *m*/*z* 1069.6 [108]. The toxic profiles by CBA-N2a of the cooked meal remnant and raw barracuda extracts were nearly identical, with eight well-defined peaks of cytotoxicity. LC-MS/MS analysis demonstrated that C-CTX-1 was the principal CTX associated with cytotoxic peak 4, contributing ~60% of the composite toxicity in these two samples [108]. Patterns of ions characteristic of CTXs were observed in full-scan and product ion spectra of cytotoxic peaks 1 and 3 (~28% of the composite toxicity) and were attributed to protonated molecules at *m*/*z* 1159 and *m*/*z* 1143 (possible C-CTX-3/-4 [120]), respectively. The authors suggested that the C-CTX congener with a [M+H]^+^ at *m*/*z* 1159 could be either a hydroxylated C-CTX-1 or an oxidised form of C-CTX-1143. Among the minor cytotoxic peaks, only peak 2 full-scan and product ion spectra were suggestive of CTX, with an abundant ion at *m*/*z* 1139 resulting from the loss of a water molecule from the protonated molecule at *m*/*z* 1157 (possible oxidised form of C-CTX-1 derivatives) [108]. The characteristic ciguatoxin MS spectra could not be confirmed for peak 6, although it accounted for 11% of the composite toxicity [108].

The same approach also confirmed that invasive lionfish species (*Pterois volitans*) caught in the waters of the U. S. Virgin Islands [39] and Saint Barthélemy (French Antilles) [174] accumulated C-CTX-1. A 12% prevalence rate of ciguatoxic lionfish from the U. S. Virgin Islands, exceeding the FDA guidance level of 0.1 ppb C-CTX-1 eq., was established using CBA-N2a, and fish size did not correlate to toxicity level [39]. Selected reaction monitoring using LC-MS/MS confirmed the presence of C-CTX-1 in CBA-N2a-positive samples compared to reference standards [39]. In lionfish from Guadeloupe or Saint-Martin, no CTX-like activity was found, and C-CTX-1 was structurally confirmed using HPLC-MS/MS with a C-CTX-1 reference standard in the eight ciguatoxic samples from Saint-Barthélemy with composite cytotoxicities ranging from 0.157–0.33 ppb P-CTX-1 eq. [174]. Among the 77 samples of fish species from the U. S. Virgin Islands tested by Loeffler et al. [37], 39 had cytotoxic activity determined using CBA-N2a. Among these, only 13 samples belonging to two fish species, red hind *Epinephelus guttatus* (*n* = 12) and white grunt *Haemulon plumierii* (*n* = 1), contained levels of C-CTX-1 detectable using LC-MS/MS. Although queen triggerfish (*Balistes vetula*) exhibited the highest cytotoxicity using CBA-N2a (0.01 to 0.11 ppb C-CTX-1 eq.), surprisingly, the presence of C-CTX-1 could not be confirmed using LC-MS/MS [37]. This suggests the possibility of potential false positive results from the CBA-N2a assay, or the presence of uncharacterised CTXs, such as the recently identified C-CTX-5 produced by the Caribbean *Gambierdiscus* species [43].

- Confirmation of C-CTX-1 and identification of C-CTX congeners in Fish from the Eastern Atlantic

In 2010, the presence of C-CTX-1 in several amberjacks (*Seriola* spp.) responsible for CP in the Eastern Atlantic (Canary and Madeira islands) was confirmed using CBA-N2a and HPLC-MS/MS, with reference to a standard of C-CTX-1 [106]. Otero et al. analysed fish implicated in several intoxications captured in the waters of the Selvagens Islands (Madeira archipelago) using ultra-high-performance liquid chromatography mass spectrometry (UHPLC-MS). The presence of ciguatoxins in a 70 kg greater amberjack *Seriola dumerili* and a 20 kg lesser amberjack *Seriola fasciata* was confirmed [107]. The biological activity of these samples was checked using an electrophysiological assay (perforated patch-clamp recording), and an analytical UHPLC-MS method was optimized using a Waters BEH C18 column (100 × 2.1 mm, 1.7 µm) eluted with a water/acetonitrile gradient containing 50 mM of formic acid and 2 mM of ammonium formate. CTX identification was achieved using an ESI-triple quadripole (Xevo TQ MS, Waters), first using a scan mass mode (*m*/*z* 1000–1500), followed by selected ion monitoring (SIM) of the two more prominent ions [M+H]^+^ with a typical fragmentation pattern of CTXs (sodium, ammonium, and proton adducts, and loss of water molecules). In this study [107], six congeners of CTX were recovered, with the three most abundant peaks detected at *m*/*z* 1040.6 (two putative CTX-3C group toxins, e.g., 51-OH-CTX-3) and at *m*/*z* 1141.6 (C-CTX-1/-2 or I-CTX-1/2). Low levels of P-CTX-1, CTX-3C, and CTX-4A/B, or an analogue at *m*/*z* 1061.0, were reported. However, in this study, references to CTXs were only available to confirm the presence of P-CTX-1 and CTX-3C [107].

Single reaction monitoring of C-CTX-1 sodium adducts as parent and product ions used for C-CTX-1 quantification (see Section 5.2.2) achieved high sensitivity in MS detection but compromised the confirmation of diagnostic fragment ions in a multiple reaction monitoring (MRM) mode [201]. Therefore, as part of the European project “EuroCigua”, further LC-MS/MS analysis was needed, using a mobile phase of aqueous acetonitrile with ammonium formate and formic acid modifiers to enhance C-CTXs precursor ions [M+H–H_2_O]^+^ and generate additional fragment ions with the aim of confirming the presence of C-CTX-1 and identifying potential CTX analogues in fish from Macaronesia [110,175,176]. The use of ammonium formate significantly reduced matrix effects, allowing for larger injection volumes without compromising detection using an ESI-triple quadrupole (6495 Agilent) [110]. C-CTX-1 was confirmed in several fish species from the Atlantic coasts of Spain and Portugal, along with C-CTX analogues (Table 5), using multiple reaction monitoring (MRM) from different types of precursor ions depending on C-CTX and corresponding to [M+H–nH_2_O]^+^, [M+H]^+^, [M+Na]^+^, or [M+H–CH_3_–H_2_O]^+^ [110,175,176].

Estevez et al. identified four C-CTX analogues showing CTX-like activity using CBA-N2a in the HPLC-purified fractions of a *Seriola fasciata* implicated in CP [175]. In this amberjack, the C-CTX congeners detected in order of increasing polarity were: C-CTX-1157, C-CTX-1 (the main analogue responsible for the CBA-N2a toxicity), C-CTX-1127, and a C-CTX-1 isomer (presumed to be C-CTX-2) [175]. All the identified C-CTXs analogues produced the characteristic ions associated with CTXs, including [M+H]^+^, [M+NH_4_]^+^, [M+Na]^+^, [M+K]^+^, and [M+H–nH_2_O]^+^ [175]. C-CTX-1157 was also reported in a zebra seabream (*Diplodus cervinus*) from the Selvagens Islands that showed composite toxicity using CBA-N2a, although C-CTX-1 was not detected using LC-MS/MS [176]. Interestingly, some of these CTX analogues had [M+H]^+^ *m*/*z* and characteristic fragments identical to those previously found in fish from the Caribbean Sea, including ions [M+H]^+^ at *m*/*z* 1157 [23,41,108] and [M+H]^+^ at *m*/*z* 1127 [23,41]. In fractions found to have CTX-like activity (CBA-N2a) but devoid of the ion corresponding to C-CTX-1 sodium adduct [M+Na]^+^ at *m*/*z* 1163.7, a new putative hydroxyl metabolite of C-CTX-1 was identified by its prominent sodium adduct at *m*/*z* 1181.7, in a study of fish from Macaronesia [110]. In addition, a product ion analysis at high collision energies of [M+H–H_2_O]^+^ *m*/*z* 1123.6 ion of C-CTX-1 showed the presence of prominent ions at *m*/*z* 108.9 and *m*/*z* 191.1 (specific fragments) [110]. Based on this result, Estevez et al. suggested a revised molecular structure of C-CTX-1 [110], that was first described in 1998 [35], arguing that the N-ring of C-CTX-1 is more likely a seven-membered ring rather than a six-membered ring (see Figure 1). However, this proposed structure was based on inconclusive MS data and not supported by NMR [35] and chemical evidence [35,43,120,202,203].

#### 5.2.4. Multianalytes Screening (P-CTXs, I-CTXs, and C-CTXs) Using HPLC-MS/MS with MRM on Precursor Ions [M+Na]^+^ and Confirmation Using MRM on Precursor Ions [M+NH_4_]^+^ (Methanol/Acetonitrile Elution)

Spielmeyer et al. [180] adopted an analytical strategy based on a partially validated new extraction protocol (see Section 4.2. and Figure 4) coupled to CBA-N2a, LC-MS/MS with a QTrap 6500+ (Sciex), and confirmation using low-resolution or high-resolution LC-MS/MS via ammonium adducts. For the two types of analyses, liquid chromatography was performed using a Gemini NX-C18 (150×2 mm, 3 µm; Phenomenex), and the eluents were (A) 1 mM ammonium acetate and 0.5% formic acid in water, and (B) methanol/acetonitrile (3:1). The multianalytes screening was developed to monitor the sodium adducts [M+Na]^+^ of >30 CTX congeners classified into four groups, CTX-4A and CTX3C groups (for P-CTXs), a C-CTX group, and an I-CTX group, but using only P-CTX-1, P-CTX-2, P-CTX-3 and P-CTX-3C standards [180]. MS confirmation was achieved by monitoring four predicted MRM transitions per congener, choosing the ammonium adduct [M+NH_4_]^+^ as the precursor ion and the corresponding [M+H]^+^ and [M+H–nH_2_O]^+^ with *n* = 1–3 as product ions [180]. Using this semi-targeted LC-MS/MS approach, 52 tissue samples of *Lutjanus bohar* all tested positive within the CBA-N2a, which revealed the presence of a complex CTX contaminant profile with several congeners of the CTX3C-group [34]. Comparing only the peak areas of the detected CTXs (without quantification), the toxin profile of the Western Indian Ocean samples (seven samples associated with an outbreak in Europe in 2020, declared as captured off the southwest coast of India) was indistinguishable from the CTX profile of the Western Pacific Ocean samples (45 samples associated with an outbreak in Europe in 2017) [34]. However, additional studies are required to confirm that the challenges in independently certifying the location of the captured fish did not contribute to the surprising disparity in the type of CTX identified.

### 5.3. Contribution of High-Resolution Mass Spectrometry for Identification and Chemical Characterisation of C-CTXs

#### 5.3.1. Contribution of HRMS to Study Fish C-CTXs

HRMS provides an opportunity for targeted analysis of C-CTX-1 and known putative analogues, as well as untargeted analysis to determine ciguatoxin diversity and new potential metabolites [113,120,131,204,205]. Acquisition in full-scan MS mode and targeted MS/MS mode using a Q-TOF 6550 iFunnel (Agilent Technologies) of C-CTX-1 reference material showed a predominant ion [M+H–H_2_O]^+^ at *m*/*z* 1123.6184 (−4.5 ppm), followed by the sodium adduct [M+Na]^+^ at *m*/*z* 1163.6106 (−1.6 ppm) [204]. The protonated C-CTX-1 and its ammonium and potassium adducts were also present, but with a lower intensity and with higher mass differences (>10 ppm) [204]. The presence of C-CTX-1 was confirmed in a naturally contaminated barred hogfish (*Bodianus scrofa*) caught near the Selvagens Islands (Portugal) due to its retention time, the detection of the first water loss [M+H–H_2_O]^+^, and the sodium adduct [M+Na]^+^. However, the sensitivity was not sufficient to obtain MS/MS spectra in this sample [204]. In another work on fish specimens from the Madeira and Selvagens Archipelagos, a HRMS full-scan methodology was used to detect putative CTXs using four criteria: *m*/*z* ratio, mass accuracy (ppm), the ring double-bond equivalent (RDBE), and the mono-isotopic pattern (M + 1 ion) of the main, with a M + 1/M ion ratio = 0.6–0.7 [113]. This method identified C-CTX-1 in the flesh of moray eels (*M. helena*, *M. augusti* and *G. unicolor*) and their corresponding livers, which exhibited composite toxicity up to six-times higher than that of the flesh. C-CTX-1 was also identified in four liver samples, with an exact mass for the [M+H]^+^ ion of 1141.6305 [113]. Tudó et al. also reported the presence of a possible CTX analogue in some flesh and/or liver samples corresponding to putative dihydro-CTX2 (C_60_H_84_O_18_ MW 1092.5652 Da) or a structural isomer, a putative new C-CTX-1109 identified by its ammonium adduct at *m*/*z* 1127.6023, as well as gambieric acid A [113]. However, further studies on the CTX-like activity of C-CTX-1109 are required to confirm its contribution to ciguatera risk.

Recently, Estevez et al. developed an analytical approach using capillary LC-HRMS to determine the ciguatoxins present in contaminated fish [131]. Full MS-data dependent acquisition mode was used for identification and quantitation of CTXs, with parallel reaction monitoring (PRM) used to obtain additional structural inferences with reference to C-CTX-1. The analyses confirmed that C-CTX-1 was the principal ciguatoxin present in four fish originating from the Canary Islands (Spain) or the Selvagen Islands (Madeira archipelago, Portugal): an amberjack (*Seriola* sp.), a cubera snapper (*Lutjanus cyanopterus*), a barred hogfish (*Bodianus scrofa*), and a dusky grouper (*Epinephelus marginatus*). Minor C-CTX analogues were also identified using full MS mode from *m*/*z* 1000–1200 Da, looking for ion patterns matching cyclic polyethers including the ciguatoxins ([M+H–nH_2_O]^+^, [M+H]^+^, [M+NH_4_]^+^, [M+Na]^+^, and [M+K]^+^. Methoxylation with formic acid was also used to assess the possible transformation in their methoxylated derivatives and to confirm the structure of their N-ring [121]. The putative structures of C-CTX analogues characterised by MS include [131]:

- 17-hydroxy-C-CTX-1 ([M+H]^+^ *m*/*z* 1157.6255) with a possible location of the -OH group in the E-ring,
- two N-seco- forms of C-CTX-1 ([M+H]^+^ *m*/*z* 1143.6462) corresponding to C-CTX-1-reduced forms, previously known as C-CTX-3/-4 [120],
- 50,51-didehydro-C-CTX-3 ([M+H]^+^ *m*/*z* 1141.6306),
- 17-hydroxy-50,51-didehydro-C-CTX-3 ([M+H]^+^ *m*/*z* 1157.6255).

The methylated analogues of C-CTX-1 and C-CTX-3/-4 were likely artefacts arising from the use of methanol and formic acid during the HPLC fractionation [121,130]. Similarly, elevated levels of C-CTX3/4 and the methylated compounds can potentially occur under acidic conditions [131]. C-CTX-1 was identified as responsible for more than 60% of the total toxin content, followed by putative 17-hydroxy-C-CTX-1, initially designated as C-CTX-1157 and identified as toxic using RBA (see Section 5.1.2) [42]. Its toxicity was confirmed using CBA-N2a [108]. The thermal stability of C-CTX-1 and 17-hydroxy-C-CTX-1 was also confirmed by cooking one of the samples.

UHPLC-HRMS/MS can also contribute structural insights from identified MS fragment ions generated chemically, including those that modify the A and N rings of C-CTX-1. Kryuchkov et al. reported that the fragmentation of the ladder-frame backbone of C-CTX-1/-2 followed a characteristic pattern and defined a generalized nomenclature for the MS/MS product ions [120]. This was aligned with previous studies that identified only three types of product ions that were formed during fragmentation of the polyether backbone [32,33,38,42]. In addition, a MS-based methodology was proposed for the characterisation of new C-CTXs, with oxidation and reduction reactions used to assist the examination of the structural modifications of C-CTX-1 found in king fish (*Scomberomorus cavalla*) and great barracuda (*Sphyraena barracuda*) samples [120]. The reduction of C-CTX-1/-2 using sodium borohydride (NaBH_4_) and borodeuteride (NaBD_4_) generated pairs of isomers with protonated molecules at *m*/*z* 1143.6463 and 1144.6535, respectively [120]. This led to the structural characterisation of two new analogues, C-CTX-3 and C-CTX-4, containing an open N-ring, and thus corresponding to C-CTX-1 and -2 reduced at C-56 [120]. Caribbean CTX congeners with a protonated molecule at *m*/*z* 1143.6 were identified earlier in several fish from the Caribbean [23,38,41,42,108], and more recently from the Eastern Atlantic Ocean [131]. However, these are an open (unstable) form of C-CTX-1 that are generated under acidic conditions and lack stereochemistry, making the assignment of the two isomers controversial. Kryuchkov et al. also showed that a pentafluorophenyl-propyl UHPLC column (100×2.1 mm, 1.7 μm) significantly improved the shape of C-CTX-1 and -2 peaks and provided better chromatographic separation between C-CTX-1/-2 and C-CTX-3/-4. However, the octadecylsilane-based LC column (100 × 2.1 mm, 1.5 μm) allowed for the best anomer separation regardless of chromatographic conditions [120]. A comparison of the peak shapes of C-CTX-1 and -2 during LC-HRMS in acidic and neutral mobile phases on the C-18 column were indicative of a rapid on-column interchange between C-CTX-1 and -2 that was accelerated under acidic conditions, whereas the peak shapes of 3 and 4 were unaffected [120].

To improve the HRMS sensitivity compared to that of targeted LC-MS/MS, Kryuchkov et al. also proposed a promising chemical derivatization approach employing a fast and simple one-pot derivatization with Girard’s reagent T (GRT) [205]. The sensitivity of their LC-MS/MS method using a triple quadrupole (6495B, Agilent Technologies, Santa Clara, CA, USA) and the LC-HRMS method on a Q-Orbitrap (Q-Exactive, Thermo Fisher Scientific, Waltham, MA, USA) was improved for C-CTX-1 by ~40- and 17-fold, respectively [205]. Derivatization with GRT improved method selectivity and ionization efficiency by inserting a quaternary ammonium ion onto C-CTX-1. However, the use of GRT derivatization for routine applications still requires validation [205].

UHPLC-HRMS methods in negative and positive ionisation modes on a Q-Orbitrap (Q-Exactive, Thermo Fisher Scientific) were also adapted for CYP and UGT metabolism experiments and chemical reduction by Gwinn et al. They reported the production of phase II GlcA conjugates of C-CTX-1/-2 in vitro by liver microsomes of five fish species from the northern Gulf of Mexico and Atlantic salmon, which was the first time it was observed [165]. Borohydride reduction of C-CTX-1/2-GlcA metabolites confirmed that the metabolites were comprised of four distinct glucuronide products, with the sugar attached at two separate sites on C-CTX-1/-2, excluding the C56 hydroxyl group as the conjugation site [165].

#### 5.3.2. Contribution of HRMS to Study *Gambierdiscus* spp. Toxins

The HRMS technique has also been applied to study algal toxin profiles [43,73,86,109,206,207]. Combining LC-MS/MS for rapid screening with a quantitative estimation and LC-HRMS for confirmation and characterisation, Estevez et al. showed that the toxin profiles of *Gambierdiscus* spp. dinoflagellates from the Mediterranean Sea and Northeast Atlantic were similar to those detected in CP endemic regions, except for the presence of CTXs, with the detection of 44-methylgambierone, gambieric acid C and D, gambierone analogue, and gambieroxide in *Gambierdiscus australes* strains (Balearic Islands, Spain) [207]. This study highlighted species and regional specificity of *Gambierdiscus* spp., with a 44-methylgambierone and gambierone-producing *Gambierdiscus* sp. strain from Crete (Greece) and an MTX-4 producing *G. excentricus* strain from the Canary Islands (Spain) [207]. HRMS did not detect maitotoxin-1 (MTX1), desulfo-MTX1, and didehydro-desulfo-MTX1 above the detection limit in Mediterranean strains of *G. australes* [207]. Sulfo-gambierone and dihydrosulfo-gambierone, two new analogues of gambierone, were also characterised in two *G. excentricus* strains initially isolated from the Bahamas and the Florida Keys [208], however, no algal ciguatoxins were detected in these two strains, which was consistent with the low ciguatoxin-like content evaluated using the CBA-N2a assay [208]. An m-aminophenylboronic acid agarose gel was used to confirm the presence of gambierone and identify a novel isomer of 44-methylgambierone in an extract of *Gambierdiscus silvae* from the U.S. Virgin Islands [206].

Recently, a major breakthrough was achieved with the identification of an algal toxin C-CTX-5 that was extracted from a *Gambierdiscus silvae* strain and two *Gambierdiscus caribaeus* strains. The toxin was isolated from macroalgae collected from reefs surrounding St. Thomas (U. S. Virgin Islands) [43]. These *Gambierdiscus* strains showed C-CTX-like activity on voltage-gated sodium channel using CBA-N2a, with a higher toxicity for *G. silvae* compared to the two *G. caribaeus* strains tested [43]. The toxins were screened for known CTXs using reference standards (C-CTX-1, gambierone and 44-methylgambierone) and unknown putative CTXs using LC-HRMS on a Q-Orbitrap (Q Exactive, Thermo Fischer Scientific) with a heated electrospray ionization probe (HESI-II) [43]. With a full-scan acquisition (*m*/*z* 1000–1250), product-ion spectra using targeted parallel reaction monitoring (PRM) scan mode, and chemical transformations, Mudge et al. identified a novel C-CTX analogue, C-CTX-5, for the first time from Caribbean *Gambierdiscus* strains [43]. An initial assessment of CTX-like activity was made using in vitro CBA-N2a, which confirmed that C-CTX-5 was ciguatoxic, with an IC_50_~½ IC_50_ of C-CTX-1/-2. However, this remains to be quantitatively evaluated using quantified purified materials [43]. Moreover, C-CTX-1/-2 were also detected compared with a reference standard, and it was estimated at ~7% of the level of C-CTX-5 in *G. silvae*. Therefore, it could also contribute directly to toxicity of fish that ingest *Gambierdiscus* cells [43]. Gambierone was detected in all algal extracts, and 44-methylgambierone was detected in *G. silvae* and one of the two *G. caribaeus* [43]. Interestingly, the transformation of C-CTX-5 into C-CTX-1/-2 occurred via a reduction step [43] rather than oxidation, as observed for Pacific toxins, CTX-4A, CTX-4B, and CTX-3C, which are oxidized to the corresponding CTXs found in fish from the Pacific Ocean [90].

## 6. Discussion and Perspectives

### 6.1. Bioassays

The cytotoxicity assay CBA-N2a reflects the overall toxicity attributed to sodium-channel blocking toxins and is currently used as an initial screening method for toxic fish. It provides an alternative to the mouse bioassay and the use of radiolabeled molecules. However, in most studies, higher values for composite toxicities were obtained compared with C-CTX-1 concentrations in fish flesh samples [37,109,110,112,155,175,176]. Discrepancies between composite cytotoxicity evaluated using CBA-N2a and concentrations of C-CTX-1 evaluated using LC-MS/MS could be due to the presence of other C-CTX congeners. Therefore, C-CTX-1 was not the principal toxin. These results emphasize the challenges of combining biological and/or functional and/or pharmacological assays with analytical methods, considering their different specificities and sensitivities. The possibility that the CBA-N2a assay can overestimate ciguatera risk needs to be investigated. Radiolabeled ligand binding assays can also be used to control fish toxicity and monitor toxic LC fractions of slow- and fast-acting C-CTXs [41,172,194]. However, the CBA-N2a assay was 12-fold more sensitive to detecting C-CTX-1 than the radiolabeled RBA [194]. It was also shown to be more sensitive than LC-MS/MS and fRBA using fluorescent brevetoxin (BODIPY^®^-PbTx-2) in a comparative study on P-CTXs from *Gambierdiscus polynesiensis* [83]. P-CTX-1 was 5–10-fold more potent than C-CTX-1 using RBA [172]; in agreement with the fact that P-CTX-1 was 14 times more toxic to mice than C-CTX-1 [38]. Thus, although it is no longer recommended by the European Food Safety Authority, the mouse bioassay could allow for the estimation of the relative toxicity of new analogues compared with C-CTX-1 or P-CTX-1 which are more accessible. This is important considering that differences in ADME processes may influence in vivo toxicity. To accelerate screening and address species differences in CTX sensitivity, Lewis et al. established a new FLIPR-based screen using human SH-SY5Y cells sensitised using ouabain and veratridine [81]. This assay demonstrated comparable sensitivity to the CBA-N2a assay, and could differentially detect MTX and CTX in extracts of *Gambierdiscus* spp. [81].

### 6.2. Considerations for Analytical Approaches for C-CTX Detection and Quantification

Significant impediments to the study of ciguatera include the scarcity of pure ciguatoxins standards [129,130] and the number of structural analogues contributing to the toxicity of hazardous fish [6,114,118,197]. The lack of sufficient quantities of pure CTX standards impedes the development and harmonization of methods for their detection and public health protection. These difficulties are compounded by the commensurately low toxin thresholds for human poisoning and a sufficient sample pretreatment to reduce matrix interferences. Typically, ultra-trace analysis of lipophilic analytes (<1 ppb) is heavily influenced by the matrix involved, with biological matrices such as fish tissues often the most challenging. Although it is time-consuming and expensive, the only way to ensure potential biological activity of new C-CTX congeners is to carry out accurate chromatographic fractionation and to test for biological activity using adapted bioassays [118,170,195,196,198].

The selection of extraction and purification procedures depends on the specific objectives of the study. Given these diverse needs, a wide range of accelerated and comprehensive protocols have been developed, which are tailored to address specific matrix issues (e.g., fish tissue, microalgae, or blood). The desired outcomes include isolating reference materials, investigating fish toxin profiles, ensuring fish safety, or facilitating clinical diagnosis. Currently, no extraction protocol from biological matrix has been validated for CTXs, regardless of their origin. Various extraction and purification methodologies (see Section 4) are used for C-CTXs [118]. Currently, extraction procedures have been adapted from the method of Lewis [195] and the FDA method developed by Dickey [209]. Studies comparing the extraction of cooked and raw samples of flesh of C-CTX-containing fish have revealed only slight differences in toxin profiles and contents [23,91,104,108,131]. Therefore, within the fish flesh matrix, both slow and fast-acting ciguatoxins appear to be heat stable. However, particular attention should be paid during sample purification of certain fish species, such as moray eel and barracuda, due to the possible presence of fast-acting toxins (FATs). Some FATs have been characterised by specific symptoms in mice, and generally include more polar compounds than C-CTX-1 [38,41,104]. Potential FATs with molecular masses <900 Da, which are more comparable to the brevetoxins (850–900 Da) than known ciguatoxins (1100–1200 Da), were suspected to contribute to the total toxicity of a highly ciguatoxic barracuda [40,41]. For this particular fish species, purification using Florisil^®^ could be deleterious [104]. Additionally, losses of toxicity in the mouse bioassay observed during TSK gel filtration suggested that these losses were due to the disappearance of FATs [41]. The chemistry of the various chromatographic resins or solvents used is crucial for FAT stability. A consensus method should be established for the extraction and purification of C-CTXs from fish samples, considering the evaluation of pre-analytic efficiency and fish species-dependent matrix effects in mass spectrometry analyses. This will enable the comparison of studies on toxin profiles in ciguatoxic fish. Currently, several scientific groups in Europe and the USA are working on C-CTXs. Thus, an interlaboratory study could be useful in validating a unique protocol, or at least for the main stages of extraction and purification (e.g., the order of extraction and defatting or the type of SPE sorbents).

The chromatographic separation stage must be optimized to minimize the co-elution of the molecules of interest. The use of UHPLC systems and the availability of numerous stationary phases contributes to improving the resolution between analytes peaks, detection sensitivity, shorter analytical run times, and reduced solvent consumption [83]. Moreover, sub-2-µm solid-core particles could enable large increases in efficiency. However, it is crucial to implement an efficient preanalytical step to eliminate interfering substances present in fish flesh, such as lipids, which can affect sample loading, MS sensitivity, and column life. The most commonly used stationary phase is reversed-phase C18 (octadecylsilane), but some authors have also used a pentafluorophenyl-propyl stationary phase to significantly improve the separation of C-CTX-1/-2 and C-CTX-3/-4 peaks [120].

Since the 2010s, three methodological approaches in low-resolution LC-MS/MS using single (SRM) or multiple reaction monitoring (MRM) modes have been distinguished (Table 6). These approaches depend on the type and sensitivity of the mass spectrometer, as well as the intended purpose, such as quantifying C-CTX-1 with a purified standard or identifying C-CTXs based on their MS characteristic pattern of ion formation. Currently, for sensitive C-CTX-1 quantification, a water/methanol gradient is combined with a SRM of the prominent adduct [M+Na]^+^ as precursor and product ions, using an ESI-triple quadrupole [109,110,112,155,175,176], which has been previously used for P-CTXs [30,210]. Confirmation of C-CTX-1 and search for C-CTX analogues are performed using a water/acetonitrile gradient and MRM using the most prominent peak of each analogue [M+Na]^+^, [M+H–H_2_O], [M+H]^+^, or [M+H–CH_3_–H_2_O]^+^, and serially dehydrated ions [110,175,176]. However, a comparison with the approach based on SRM of sodium adduct as precursor and product ions [30] showed that using an ESI-triple quadrupole/linear ion trap (API 4000, Sciex) and a methanol-based mobile phase, better selectivity through confirmatory transitions and better sensitivity with less matrix effects allowed for the detection of P-CTX-1 and P-CTX-3C by choosing [M+NH_4_]^+^ and [M+H]^+^ ion precursors, respectively, and [M+H–2H_2_O]^+^ as product ions [211].

A third approach was developed for multi-analytes screening of CTXs sorted into four groups: CTX-4A and CTX3C groups (for P-CTXs), a C-CTX group, and an I-CTX, by monitoring sodium adducts separated with a water/methanol/acetonitrile gradient [180]. MS confirmation was achieved by monitoring four predicted MRM transitions per CTX and choosing the ammonium adduct [M+NH_4_]^+^ as the precursor ion and the corresponding [M+H]^+^ and [M+H–nH_2_O]^+^ with *n* = 1–3 as product ions [180]. This approach allowed for the relative contribution of the identified ciguatoxins from the CTX3C group to be compared using LC-MS/MS in 52 tissue samples of *Lutjanus bohar* [34]. This approach could be extended to species-specific CTX metabolism and inter-oceanic CTX toxin profile analyses. Indeed, analytical validation performed on three different fish genera (*Lutjanus*, *Scarus*, and *Epinephelus*) also highlighted quantitative species-specific differences in MS responses, suggesting fish-specific matrix effects, which is a major problem for quantification from different matrices [180]. Thus, another promising perspective is the synthesis of the ^18^O-labeled C-CTX-1/2, C-CTX-3/4, gambierone, and 44-methylgambierone quantified by Mudge et al. [132]. Importantly, isotope-labeled standards allow for isotope dilution mass spectrometry and gold-standard correction for matrix effects and analyte losses during sample preparation.

In addition to the targeted mass spectrometry approach using (U)HPLC-MS/MS, high-resolution mass spectrometry (HRMS) represents a promising advance for the structural identification of marine biotoxins by improving the accuracy of assigning chemical formulas [212]. However, HRMS instruments such as the Quadrupole Time-of-Flight (QToF) and Q-Orbitrap are expensive and can generate large amounts of complex data, which is not always convenient for routine analysis. Moreover, HRMS is usually less sensitive than targeted LC–MS/MS and is insufficient to quantify CTX levels in the sub-ppb range. In the study of Sibat et al., HRMS sensitivity was 2- to 12-fold lower for P-CTXs, but with an increased mass accuracy, the presence of more concentrated P-CTX analogues was confirmed, and interferences determined by co-eluting compounds was also identified [211]. Some LC-HRMS studies have confirmed the presence of targeted C-CTX-1 when reference materials are available [113,120,131,204,205] and have provided structural information on new C-CTX analogues [113,120,131,205]. LC-HRMS remains a complementary technique. Due to its cost and complexity, utilizing this method requires significant investments and technical skills, making it inaccessible to many Caribbean countries where the control of ciguateric fishes is necessary.

### 6.3. Multiplicity of Caribbean Ciguatoxins

A key point emerging from the literature is the presence of multiple Caribbean ciguatoxins in fish from the Atlantic Ocean and Caribbean Sea. This multiplicity, reminiscent of P-CTX multiplicity [33,119], could be due to strain-dependent variations in toxin content of dinoflagellates belonging to the genus *Gambierdiscus*, as well as differences in the metabolic activities of fish, especially in their microsomal detoxication capacities including glucuronidation [165]. For decades, research has focused on ciguatera fish poisoning in the Lesser and Greater Antilles, as well as Southern Florida. Since 2004, fish caught in the Northeast Atlantic (Canary Islands and Archipelago of Madeira) have been associated with ciguatera-type poisoning. So far, no P-CTXs have been identified in fish from this new ciguatera area. To date, C-CTX-1 (C_62_H_92_O_19_, 1140.6 Da) and occasionally its isomer C-CTX-2 have been found in fish from the Caribbean Sea ([22,23,35,37,38,39,41,42,108,172,174,200], and now from the Canaries Islands and Madeira Archipelago [109,110,112,130,131,155,175,176].

Only C-CTX-1 (1140.6 Da) and one of its isomers, C-CTX-2, have been structurally characterized using NMR. Their in vivo toxicity in mice is known, with lethal doses of 3.6 and 1 µg·kg^−1^, respectively, and their mode of action has been demonstrated. They target receptor site 5 of voltage-gated sodium channels. Few studies relating to the toxin concentrations of C-CTX-1 have been carried out with a calibration, using a standard of C-CTX-, due to limited amounts of C-CTX-1-purified material [23,41,42,131,200]. Lewis at al. estimated that ~50% of the toxicity determined using the MBA was explained by the presence of C-CTX-1 in 30 fish from the Caribbean [200]. Thus, C-CTX-1 may not be the only biomarker that can confirm the presence of C-CTXs in fish flesh, as C-CTX-1 and other C-CTX analogues have been detected in fish from the Lesser Antilles [23,37,38,39,41,42,108] or the Northeast Atlantic ocean [109,110,131,175]. By using HRMS combined with chemical reactions, some C-CTX congeners have been tentatively characterised (see Section 5.3.1). The congeners C-CTX-1159, C-CTX-3/-4, C-CTX-1157, and C-CTX-1127, were identified in fractions that showed toxic activity either using CBA-N2a or RBAs (Table 7). However, due to their similar structures, they are difficult to separate in reverse-phase LC, posing a challenge in confirming their toxicity with certainty.

Finally, among the analogues, two groups of C-CTX congeners have been recurrently reported in fish from the Atlantic zone:

- Compounds with [M+H]^+^ *at m*/*z* 1143.6: isomers previously known as C-CTX-1143 showed no significant activity in radio-ligand binding assays [23,41]. However, RBA may not be sensitive enough to detect low amounts in fish, and another study identified an analogue, C-CTX-1143, using LC-MS/MS in a positive fraction by CBA-N2a [108]. Two anomers, C-CTX-3 and C-CTX-4 (C_62_H_94_O_19_), have been characterised using HRMS as the reduced forms of C-CTX-1 [120,131]. Their toxic potential and their role in fish total toxicity are yet to be clearly demonstrated due to their unstable hemiketal structure.
- Compounds with [M+H]^+^ at *m*/*z* 1157.6: two compounds with [M+H]^+^ at *m*/*z* 1157.6 were recently identified using LC-HRMS, 17-hydroxy-C-CTX-1 ([M+H]^+^ *m*/*z* 1157.6255) with a possible location of the -OH group in the E-ring, and putative 17-hydroxy-50,51-didehydro-C-CTX-3 ([M+H]^+^ *m*/*z* 1157.6255) [131]. An analogue C-CTX-1157 was also reported in a CTX-positive fraction analysed using CBA-N2a. [108] and in a positive fraction analysed using RBA (unpublished data from [40]).

C-CTX profiles may vary depending on the fish species and their regional distribution. A notable observation from studying toxin profiles [23,41,42,108,131] is that in the most toxic fish, at the limit of edibility or inedible, C-CTX-1 appears to be predominant. Among the 12 samples of *Caranx latus*, the highest relative levels of C-CTX-1 (~75–90%) were observed in low toxic specimens. Their slight losses of activity during the purification stage indicated that unstable CTXs were not present [42]. In contrast, highly toxic horse-eye jacks had relatively low levels of C-CTX-1 (~50%) and were subject to larger toxicity losses upon purification, indicating that relatively high levels of unstable CTXs were present [42]. In a highly ciguatoxic barracuda from Guadeloupe, two distinct fractions were purified depending on symptomatology in mice. C-CTX-1 accounted for ~90% of the total toxicity (49 ppb in first fraction). However, despite important losses in toxicity (>60%) during purification of the second fraction, peak of sodium channel activity was identified, and two pseudo-molecular ions at *m*/*z* 809.43 and *m*/*z* 857.42 were detected using LC-MS. In a barracuda associated with a CP, C-CTX-1 contributed to ~60% of the toxicity found using CBA-N2a [108]. In fish from Macaronesia, C-CTX-1 was detected in the CTX-like active fractions exhibiting the highest toxicity [130,175], which represented more than 60% of the total toxin content (0.16 to 0.56 ppb), followed by 17-hydroxy-C-CTX-1 (0.11 to 0.23 ppb) in four fish [131]. However, these results must be analysed with caution, as these studies mostly evaluated variable percentages of congeners (or groups of isomers with the same molecular weight) depending on the fish and their total toxicity [42]. Thus, the relative percentages considered all the putative C-CTXs identified through LC-MS or LC-MS/MS analyses, whereas the data on their in vitro and/or in vivo toxicity compared to that of C-CTX-1 are still lacking.

Finally, the recent discovery by Mudge et al. of C-CTX-5 (1138.6 Da) is a significant step towards an understanding of chemical ciguatera biogenesis [43]. This new algal toxin related to C-CTX-1 was found in isolates of dinoflagellates collected in St. Thomas in the U. S. Virgin Islands, a geographical area at risk in the Caribbean Sea [43]. C-CTX-5 is likely to be half as potent as C-CTX-1 based on in vitro CBA-N2a. It was demonstrated that metabolization by liver microsomes of an omnivorous fish (sheepshead *Archosargus probatocephalus*) at the C3 position produces C-CTX-1/-2 [43]. In addition, *G. silvae* appears to be capable of producing low levels of C-CTX-1 (~7–8% of the level of C-CTX-5) [43]. Thus, *Gambierdiscus silvae* and *Gambierdiscus caribaeus* can be implicitly considered as algal producers of the Caribbean ciguatera food chain. To our knowledge, no analogue with [M+H]^+^ at *m*/*z* 1139.6 has been reported in Atlantic fish. This research work [43] offers new perspectives for studying the dynamics of benthic *Gambierdiscus* CTX-producers and their conditions of production of C-CTX-5 and small amounts of C-CTX-1, as well as for the production of analytical standards based on C-CTX-5 using chemical or enzymatic reactions. It also enables the study of the C-CTXs dynamics during the Caribbean food chain transmission.

## 7. Conclusions

The recent finding that C-CTX-5, produced by *G. silvae* and *G. caribeus*, is the likely algal precursor of fish C-CTX and undergoes biotransformation to C-CTX-1/-2 in fish liver microsomes fills a significant knowledge gap in understanding the origins of Caribbean C-CTXs. This finding provides a coherent link to C-CTX-1, which typically dominates ciguatoxic fish from the Caribbean. Importantly, C-CTX-5 from *G. silvae* was confirmed to be a voltage-gated sodium channel activator, indicating its potential to contribute to ciguatera.

Despite these advances, the relative potencies of new Caribbean ciguatoxins still remains to be determined using pure and/or calibrated standards. However, until these become available, an estimation of total CTX activity based on voltage-gated sodium channel activity (ideally human sodium channels) should be used to quantify ciguatera risk when multiple contaminating congeners are present. Recent developments in LC-MS/MS allow for confirmation of C-CTXs in fish from the Caribbean Sea and Northeast Atlantic. Quantitation of C-CTX-1 is dependent on the availability of reference materials, which continue to limit analytical approaches, including the development of new extraction and purification procedures. The recent use of high-resolution mass spectrometry also provides an important contribution to chemical and toxin profile analysis in fish and dinoflagellates. However, it is not appropriate for public health controls, especially in small, under-resourced islands where ciguatera is a major public health problem. The lack of commercially available certified C-CTX reference materials is currently the most critical concern. This constitutes the main difficulty in developing an official LC-MS monitoring program for Caribbean CTXs.

Finally, since the first studies in the 1950s, ciguatera researchers initially investigated the origins, distribution, chemistry and detection of P-CTXs. Despite significant, more recent progress, Caribbean ciguatoxins remain less understood, in part due to the multiplicity and variability of the congeners involved. This is reinforced by the diversity of associated vectors, dinoflagellates, and fish species at risk, and by the variability of ciguatoxic effects and responses in humans. This complexity likely reflects differences in food chain-associated detoxification mechanisms, which are only now being studied. However, despite this complexity, the clinical features of Caribbean ciguatera have remained constant over the last 50 years, supporting the view that appropriate toxicity measures can accurately reflect ciguatera risk.

To conclude, the methodological approach used for monitoring ciguatoxins usually includes two-tiers, with a first tier using a biological assay screen and the second tier using analytical methods. Protocols that have been developed for the identification and/or quantification of CTXs were compared in this review, including basic (HPLC-MS) to more sophisticated (UHPLC-MS/MS or HRMS) approaches. Each approach has advantages, disadvantages, and contributions to the field discussed. However, to control fish toxicity within a public health framework, alternative bioassays can more simply account for the multiplicity of similar toxins that often contribute to human poisoning. This includes, for example, the use of the mouse bioassay to control the shellfish toxins (50 lipophilic toxins with a common unique backbone) responsible for paralytic shellfish poisoning (PSP), which was the reference biological method validated by the AOAC (Association of Official Analytical Chemists) [213] and used in Europe until January 2019 [214,215,216,217]. Recently, the MBA was replaced by the HPLC-fluorescence method developed by Lawrence et al., which is now the official reference method for PSP detection in the European Union [217]. Thus, presently, liquid chromatography (LC) coupled with mass spectrometry (MS) appears to be the preferred method of obtaining accurate toxin profile data for ciguatoxins, as it combines a qualitative structural approach and a quantitative analysis when standards are available. Analytical approaches also have merit in distinguishing between the three structural families of ciguatoxins, depending on the ocean. However, until standards are available for all significant forms, bioassays, including CBA-N2a and MBA, continue to play a key role in screening for ciguateric fishes.

## Figures and Tables

**Table 1:** Names, molecular formulae, molecular mass (in Da), known sources (*Gambierdiscus* spp. or fish), and structural characteristics of the three major types of ciguatoxins from the Pacific and Atlantic Oceans.

CTX	Other Names	Molecular Formula	Molecular Mass (Da)	Known Source	Structural Characteristics
Type 1 (60 carbons, A to M rings, DEF rings size 7/7/9)					
**P-CTX-1**	CTX-1B, CTX, CTX1	C_60_H_86_O_19_ [98]	1110.6	Fish	
**P-CTX-3**	54-deoxy-CTX-1B	C_60_H_86_O_18_ [122]	1094.6	Fish and *Gambierdiscus* spp.	Two epimers without any -OH group at C54, compared to P-CTX-1
P-CTX-2	52-epi-54-deoxy-CTX-1B	C_60_H_86_O_18_ [125]	1094.6		
**GT-4B** ^a^	GTX-4B, CTX-4B, 52-epi-P-CTX-4A	C_60_H_84_O_16_ [98]	1060.6	*Gambierdiscus*/*Fukuyoa* spp. and herbivorous fish	Two epimers at C52 differing from P-CTX-1 by the absence of 2 -OH on A-ring and one -OH on M-ring
CTX-4A ^a^	P-CTX-4A	C_60_H_84_O_16_ [126]	1060.6		
Type 2 (57 carbons, A to M rings, DEF rings size 7/8/9)					
**CTX-3C** ^a^	P-CTX-3C	C_57_H_82_O_16_ [123]	1022.6	*Gambierdiscus*/*Fukuyoa* spp.	Two epimers at C49
CTX-3B	P-CTX-3B, 49-epi-P-CTX-3C	C_57_H_82_O_16_ [119]	1022.6		
Type 3 (62 carbons, A to N rings, DEF rings size 7/8/9)					
**C-CTX-1**		C_62_H_92_O_19_ [35]	1140.6	Fish	Two epimers at C52
C-CTX-2		C_62_H_92_O_19_ [35]	1140.6	Fish	
C-CTX-3	C-CTX-1143	C_62_H_94_O_19_ [120]	1142.6	Fish	Two anomers of the hemiketal ring opened between C52 and C56, reduced forms of C-CTX-1/-2
C-CTX-4	C-CTX-1143	C_62_H_94_O_19_ [120]	1142.6	Fish	
C-CTX-5 ^b^		C_62_H_90_O_19_ [43]	1138.6	*G. silvae*, *G. caribaeus*	C-CTX-1 with the OH at C3 reduced to a ketone

The more energetically favourable epimers (lower energy) are shown in bold. ^a^ isolated from a *Gambierdiscus* spp. strains from French Polynesia [98,99,100,123,126]. ^b^ isolated from *Gambierdiscus silvae* and *Gambierdiscus caribaeus* strains from the Caribbean [43].

**Table 2:** Retention time, pseudo-molecular ion *m*/*z*, and name of corresponding C-CTX congeners detected using HPLC-MS [42] and radiolabeled-ligand binding (RLB) analysis (unpublished data reported from [40]) of a TSK extract from a Caribbean horse-eye jack (*C. latus*) known as CL5 [42]. Peaks A to E correspond to Na^+^ channel binding of CTX analogues (intensity levels are indicated in brackets). LC-MS analyses were conducted using a Zorbax C3 300 SB (2.1 × 150 mm) column using a water/acetonitrile gradient with 0.1% formic acid and a Quadrupole Time-of-Flight PE Sciex QStar Pulsar.

Retention Time (min)	[M+H]^+^ *m*/*z*	C-CTX-	RLB Peaks (Relative Intensity)
48.51	1159.58 859.42 * 811.47 **	1159	A (+)
51.55	1143.57	1143a	no peak
52.03	1143.60	1143	no peak
52.17	1157.57	1157	B (++)
53.20	1181.60	1181	
54.47	1141.58	1141a	
54.84	1141.58	1	C (+++)
55.26	1127.57	1127	
58.99	1141.61	2	D (+++)
60.32	1141.58	1141b	no peak
61.38	1141.58	1141c	E (+++)

* compounds identified by ions [M+H]^+^, [M+Na]^+^, [M+K]^+^, [M+H–nH_2_0]^+^ (*n* = 1–4). ** compound identified by ions [M+H]^+^ and [M+H–nH_2_0]^+^ (*n* = 1–4).

**Table 3:** Summary of analytical approaches and results of C-CTX-1 quantification using MRM with an ESI-triple quadrupole (6495 Agilent) in five studies on fish from the Macaronesia Islands. Quantitation was done using a P-CTX-1 pure standard, and LOD and LOQ were 0.0045 ppb and 0.015 ppb P-CTX-1 eq., respectively [109,110,112,175,176].

**References**	[109]	[110]	[175]	[176]	[112]
**LC conditions**					
LC column	Agilent Poroshell 120-EC C18 (50 × 3 mm, 2.7 µm)				
Mobile phases A and B	A: 5 mM ammonium formate and 0.1% formic acid in H_2_O; B: Methanol				
[M+Na]^+^ *m*/*z* for precursor and product ions in MRM for **CTXs type 1**					
1133.6 (P-CTX-1)	**+** ^#^	**+** ^#^	**+** ^#^	**+** ^#^	**+** ^#^
1117.6 (P-CTX-2/-3)	**+** ^#^	**+** ^#^	**+** ^#^	**+** ^#^	**+** ^#^
1083.6 (GT-4B/CTX-4A)	**+** ^#^	**+** ^#^	**+** ^#^	**+** ^#^	**+** ^#^
1101.6 (M-secoCTX-4B/-4A)	u.m.	**+** ^#^	u.m.	u.m.	u.m.
[M+Na]^+^ *m*/*z* for precursor and product ions in MRM for **CTXs type 2**					
1045.6 (CTX-3C/-3B)	**+** ^#^	**+** ^#^	**+** ^#^	**+** ^#^	**+** ^#^
1061.6 (51-hydroxyCTX3C)	**+** ^#^	**+** ^#^	**+** ^#^	**+** ^#^	u.m.
1063.6 (2-hydroxy-CTX3C)	u.m.	**+** ^#^	**+** ^#^	u.m.	u.m.
1079.6 (2,3-dihydroxyCTX-3C)	**+** ^#^	**+** ^#^	**+** ^#^	**+** ^#^	u.m.
1063.6 (M-seco-CTX3C)	u.m.	**+** ^#^	u.m.	u.m.	u.m.
1077.6 (M-secoCTX3C-methylacetal)	u.m.	**+** ^#^	u.m.	u.m.	u.m.
[M+Na]^+^ *m*/*z* for precursor and product ions in MRM for **C-CTXs type 3**					
1163.7 (C-CTX-1)	**+** ^#^	**+** ^#^	**+** ^#^	**+** ^#^	**+** ^#^
1177.6 (C-CTX-1-Me)	u.m.	**+**	u.m.	**+** ^#^	**+** ^#^
**Results for different fish**					
Fish sampling (fishing spot)	11 fish ^a^ (SI) 9 fish ^b^ (M)	3 fish ^c^ (CI) 1 fish ^d^ (SI)	1 fish ^e^ (SI)	56 fish ^f^ (SI)	109 fish ^g^ (CI) (flesh, liver)
Cytotoxicity levels by CBA-N2a (min–max)	not used	Positive CTX-like toxicity	1.4 ppb *	0.006–0.75 ppb **	<LOQ–1.365 ppb ** (93 flesh) <LOQ–6.4390 ppb** (107 livers)
Fish C-CTX-1 levels by LC-MS/MS (min–max)	C-CTX-1 ^#^ (8 fish, SI) <LOQ–0.25 ppb *	C-CTX-1 ^#^ (4 fish) 0.12–0.76 ppb *	C-CTX-1 ^#^ (1 fish) 0.84 ppb *	C-CTX-1 ^#^ (20 fish) <LOQ–0.48 ppb *	C-CTX-1 ^#^ (30 fish/62 analysed) 0.018–0.270 ppb *

**+** = monitored ion. u.m. = unmonitored ion. CBA-N2a = neuroblastoma cell-based assay. CI = Canary Islands (Spain). M = Madeira (Portugal). SI = Selvagens Islands (Portugal). * Expressed in C-CTX-1 equivalents (FDA guidance: 0.1 μg/kg C-CTX-1 eq. = 0.1 ppb); ** expressed in P-CTX-1 equivalents (FDA guidance: 0.01 μg/kg P-CTX-1 eq. = 0.01 ppb). ^#^ Confirmation with a reference standard. ^a^ *Epinephelus marginatus* (dusky grouper, *n* = 1), *Bodianus scrofa* (barred hogfish, *n* = 3), *Balistes capriscus* (grey triggerfish, *n* = 2), *Mycteroperca fusca* (island grouper, *n* = 1), *Serranus atricauda* (blacktail comber, *n* = 1), *Kyphosus sectatrix* (bermuda sea chub, *n* = 2), *Sphyraena viridensis* (barracuda, *n* = 1) [109]. ^b^ *Seriola rivoliana* (longfin yellowtail, *n* = 1), *Makira nigricans* (blue marlin, *n* = 1), *Dentex gibbosus* (pink dentex, *n* = 3), *Seriola dumerili* (greater amberjack, *n* = 2), *Sphyrna zygaena* (smooth hammerhead, *n* = 1), *Isurus oxyrinchus* (shortfin mako, *n* = 1) [109]. ^c^ *Epinephelus marginatus* (dusky grouper, *n* = 1, implicated in CP), *Seriola* spp. (amberjack, *n* = 1, implicated in CP); *Lutjanus cyanopterus* (cubera snapper, *n* = 1) [110]. ^d^ *Pagrus Pagrus* (red porgy, implicated in CP) [110]. ^e^ *Seriola fasciata* (amberjack implicated in CP) [175]. ^f^ *Kyphosus sectatrix* (bermuda sea chub, *n* = 5), *Aluterus scriptus* (scribbled leatherjacket, *n* = 1), *Sparisoma cretense* (parrotfish, *n* = 14), *Diplodus cervinus* (zebra seabream, *n* = 1), *Bodianus scrofa* (barred hogfish, *n* = 16), *Balistes capriscus* (grey triggerfish, *n* = 4), *Serranus atricauda* (blacktail comber, *n* = 5), *Sphyraena viridensis* (yellowmouth barracuda, *n* = 3), *Seriola dumerili* (greater amberjack, *n* = 1), *Seriola fasciata* (lesser amberjack, *n* = 2), *Seriola rivoliana* (longfin yellowtail, *n* = 2), *Seriola* spp. (amberjack, *n* = 1) [176]. ^g^ *Seriola* spp. (amberjack, *n* = 60), *Epinephelus marginatus* (dusky grouper, *n* = 27), *Muraena helena* (black moray eel, *n* = 11), *Diplodus vulgaris* (common two-banded seabream, *n* = 11) [112].

**Table 4:** Summary of analytical approaches and results of C-CTX-1 confirmation by MRM using an ESI-quadrupole-linear ion trap instrument (QTRAP 4000, Applied Biosystems) in four studies on fish specimens from the Atlantic Ocean (Lesser Antilles) [37,39,108,174].

**References**	[108]	**[39]**	[174]	**[37]**
**LC conditions**				
LC column	Phenomenex Luna C8(2) (150 × 2.0 mm)	Phenomenex Luna C18 (100 × 2 mm, 3 μm)	Phenomenex Kinetex C8 (75 × 2.1 mm, 2.6 µm)	Phenomenex Kinetex C8 (75 × 2.1 mm, 2.6 µm)
Mobile phase A	H_2_O	H_2_O	H_2_O	H_2_O
Mobile phase B	100% acetonitrile	95% acetonitrile	95% acetonitrile	95% acetonitrile
Modifier (A and B)	0.1% formic acid	5 mM ammonium acetate	5 mM ammonium formate	0.1% formic acid
**MRM transitions for C-CTX-1 detection**				
[M+H–H_2_O]^+^ *m*/*z* precursor ion	1123.6			
[M+H–nH_2_O]^+^ *m*/*z* product ions	1105.6; 1087.6; 1069.6	1105.7; 1087.7; 1069.8	1087.6; 1069.7	1105.6; 1087.6; 1069.6
**Results for different fish**				
Fish sampling (fishing spot)	1 barracuda ^a^ (UO)	153 lionfish ^b^ (VI)	60 lionfish ^b^ 55 (SB), 5 (SM), 30 (G)	77 fish ^c^ (VI)
Cytotoxicity levels by CBA-N2a	1.6 ppb * (cooked) 2.1 ppb * (raw)	<0.1 ppb * (43 fish) >0.1 ppb * (19 fish)	<0.01 ppb ** (5 fish, SB) 0.01–0.1 ppb ** (14 fish, SB) >0.1 ppb ** (8 fish, SB)	≥0.005 ppb * (29 fish) >0.1 ppb * (3 fish)
C-CTX-1 confirmation by LC-MS/MS	C-CTX-1 ^#^ (1 fish)	C-CTX-1 ^#^ (19 fish)	C-CTX-1 ^#^ (8 fish, SB)	C-CTX-1 ^#^ (13 fish)
C-CTX congeners detected by LC-MS/MS	C-CTX-1159, -1157 and -1143	C-CTX-2 ^#^	-	-

CBA-N2a = neuroblastoma cell-based assay. G = Guadeloupe (Lesser Antilles). SM = Saint Martin (Lesser Antilles). SB = Saint Barthélemy (Lesser Antilles). UO = unknown origin. VI = U. S. Virgin Islands (Lesser Antilles). * Expressed in C-CTX-1 equivalents (FDA guidance: 0.1 μg/kg C-CTX-1 eq. = 0.1 ppb). ** Expressed in P-CTX-1 equivalents (FDA guidance: 0.01 μg/kg P-CTX-1 eq. = 0.01 ppb). ^#^ Confirmation with a C-CTX-1 reference standard. ^a^ *Sphyraena* sp. (barracuda implicated in CP). ^b^ *Pterois volitans*. ^c^ *Balistes vetula* (queen triggerfish, *n* = 20), *Epinephelus guttatus* (red hind, *n* = 20), *Ocyurus chrysurus* (yellowtail snapper, *n* = 17), *Haemulon plumierii* (white grunt, *n* = 20) [37].

**Table 5:** Summary of analytical approaches and results of C-CTX-1 confirmation and C-CTX identification using MRM and an ESI-triple quadrupole (6495 Agilent) on fish specimens from the North-East Atlantic Ocean (Macaronesia) [110,175,176]. Precursor ions are in bold.

**References →**	[110]	[175]	[176]
**LC conditions**			
LC column	Agilent Poroshell 120-EC C18 (100 × 2.1 mm, 2.7 µm)		
Mobile phases	(A) 5 mM ammonium formate and 0.1% formic acid in H_2_O; (B) acetonitrile		
**Precursor and Product ions for confirmation of C-CTX-1 ^#^ and isomers**			
[M+H–H_2_O]^+^ *m*/*z* 1123.6	C-CTX-1 [M+H–nH_2_O]^+^ *m*/*z* 1105.6; 1087.6; 1069.6		
	specific fragments *m*/*z* 191.1; 108.9		
**Precursor and Product ions for confirmation of C-CTX-1157**			
[M+Na]^+^ *m*/*z* 1179.6	u.m.	[M+Na]^+^ *m*/*z* 1179.6	
[M+H–H_2_O]^+^ *m*/*z* 1139.6	u.m.	[M+H–nH_2_O]^+^ *m*/*z* 1121.6; 1103.6; 1085.6	[M+H–nH_2_O]^+^ *m*/*z* 1121.6; 1103.6; 1085.6
	u.m.	specific fragments *m*/*z* 191.1; 108.9	specific fragments *m*/*z* 191.1; 108.9
**Precursor and Product ions for confirmation of C-CTX-1127**			
[M+Na]^+^ *m*/*z* 1149.6	u.m.	C-CTX-1127 [M+Na]^+^ *m*/*z* 1149.6	u.m.
[M+H]^+^ *m*/*z* 1127.6	u.m.	[M+H–nH_2_O]^+^ *m*/*z* 1109.6; 1091.6; 1073.6; 1055.6	u.m.
**Precursor and Product ions for confirmation of C-CTX-1-Me**			
[M+H–CH_3_–H_2_O]^+^ *m*/*z* 1123.6	u.m.	u.m.	[M+H–nH_2_O]^+^ *m*/*z* 1105.6; 1087.6; 1069.6
	u.m.	u.m.	specific fragments *m*/*z* 191.1; 108.9
**Results for studied fish specimens**			
Fish sampling (fishing spot)	3 fish ^a^ (CI) 1 fish ^b^ (SI)	1 fish ^c^ (SI)	56 fish ^d^ (SI)
Cytotoxicity levels by CBA-N2a (min–max)	Positive	1.4 ppb *	0.006–0.75 ppb **
C-CTX-1 confirmation by LC-MS/MS	C-CTX-1 (4 fish)	C-CTX-1 (1 fish)	C-CTX-1 (20 fish)
C-CTX congeners detected by LC-MS/MS	C-CTX-1181 *	C-CTX-1157 *, C-CTX-1127 *, C-CTX-1 isomer *	C-CTX-1157

Abbreviations: u.m. = unmonitored ion. CBA-N2a = neuroblastoma cell-based assay. CI = Canary Islands (Spain). SI = Selvagens Islands. ^#^ Confirmation with a reference standard. * CTX-positive response (CBA-N2a). ** Expressed in P-CTX-1 equivalents (FDA guidance: 0.01 μg/kg P-CTX-1 eq. = 0.01 ppb). ^a^ *Epinephelus marginatus* (dusky grouper, *n*=1, implicated in CP) *Seriola* spp. (amberjack, *n* = 1, implicated in CP); *Lutjanus cyanopterus* (cubera snapper, *n* = 1) [110]. ^b^ *Pagrus Pagrus* (red Porgy implicated in CP) [110]. ^c^ *Seriola fasciata* (amberjack implicated in CP) [175]. ^d^ *Kyphosus sectatrix* (bermuda sea chub, *n* = 5), *Aluterus scriptus* (scribbled leatherjacket, *n* = 1), *Sparisoma cretense* (parrotfish, *n* = 14), *Diplodus cervinus* (zebra seabream, *n* = 1), *Bodianus scrofa* (barred hogfish, *n* = 16), *Balistes capriscus* (grey triggerfish, *n* = 4), *Serranus atricauda* (blacktail comber, *n* = 5), *Sphyraena viridensis* (yellowmouth barracuda, *n* = 3), *Seriola dumerili* (greater amberjack, *n* = 1), *Seriola fasciata* (lesser amberjack, *n* = 2), *Seriola rivoliana* (longfin yellowtail, *n* = 2), *Seriola* spp (amberjack, *n* = 1) [176].

**Table 6:** Summary of the main analytical conditions used for the confirmation and quantification of C-CTX-1 and the screening of new/putative CTX analogues using (U)HPLC-MS/MS in multiple reaction monitoring, as detailed in Part 5.

Review Section	Organic Eluent	Eluent Modifier	Goal (MS Mode)	Precursor Ions	References
5.2.2	Methanol	Formic acid and ammonium formate	C-CTX-1 quantification (SRM)	[M+Na]^+^	[109,110,112,155,175,176]
5.2.3	Acetonitrile	Formic acid or ammonium formate or ammonium acetate	C-CTX-1 confirmation (MRM)	[M+H–H_2_O]^+^	[37,39,108,174]
			C-CTXs analogues identification (MRM)	[M+Na]^+^; [M+H–H_2_O]; [M+H]^+^, [M+H–CH_3_–H_2_O]^+^	[110,175,176]
5.2.4	Methanol and acetonitrile (3:1)	Formic acid and ammonium acetate	4 CTX groups identification (MRM)	[M+Na]^+^	[34,180]
			CTXs confirmation (MRM)	[M+NH_4_]^+^	

**Table 7:** Caribbean ciguatoxins analogues (C-CTXs) identified in fish from the Caribbean Sea and Macaronesia. Published pseudomolecular ion [M+H]^+^ *m*/*z*, polarity, potential toxicity using radio-ligand binding assays, or neuroblastoma cell-based assay of the fraction containing C-CTX and putative structure.

Name	C-CTX-1159	C-CTX-3/-4 [120]	C-CTX-1157	C-CTX-1127
[M+H]^+^ *m*/*z*	1159.6	1143.6	1157.6	1127.6
Polarity *	+ 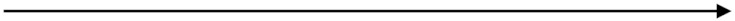 −			
Reported in Caribbean fish	[42,108]	[23,38,41,42,108,120]	[23,38,41,42,108]	[23,41,42]
Reported in fish from Macaronesia	[110]	[131]	[175,176]	[113,175]
Potential toxicity	[108,110]	[108]	[40,108]	[175]
Putative structure	Hydroxylated C-CTX-1 or oxidised C-CTX-1143	reduced C-CTX-1/-2 hemiketals [120]	Oxidised C-CTX-1	Loss of CH_2_ or demethylated C-CTX-1

* in terms of polarity, C-CTX-1 is before C-CTX-1127.

## Data Availability

Not applicable.
